# Collagen-mimetic peptide-modifiable hydrogels for articular cartilage regeneration

**DOI:** 10.1016/j.biomaterials.2015.02.079

**Published:** 2015-06

**Authors:** Paresh A. Parmar, Lesley W. Chow, Jean-Philippe St-Pierre, Christine-Maria Horejs, Yong Y. Peng, Jerome A. Werkmeister, John A.M. Ramshaw, Molly M. Stevens

**Affiliations:** a Department of Materials, Imperial College London, Exhibition Road, London SW7 2AZ, United Kingdom; b Department of Bioengineering, Imperial College London, Exhibition Road, London SW7 2AZ, United Kingdom; c Institute of Biomedical Engineering, Imperial College London, Exhibition Road, London SW7 2AZ, United Kingdom; d CSIRO Manufacturing Flagship, Bayview Avenue, Clayton, Victoria 3169, Australia

**Keywords:** Hydrogel, Mesenchymal stem cell, Biodegradation, Bioactivity, Biomimetic material, Cartilage tissue engineering

## Abstract

Regenerative medicine strategies for restoring articular cartilage face significant challenges to recreate the complex and dynamic biochemical and biomechanical functions of native tissues. As an approach to recapitulate the complexity of the extracellular matrix, collagen-mimetic proteins offer a modular template to incorporate bioactive and biodegradable moieties into a single construct. We modified a Streptococcal collagen-like 2 protein with hyaluronic acid (HA) or chondroitin sulfate (CS)-binding peptides and then cross-linked with a matrix metalloproteinase 7 (MMP7)-sensitive peptide to form biodegradable hydrogels. Human mesenchymal stem cells (hMSCs) encapsulated in these hydrogels exhibited improved viability and significantly enhanced chondrogenic differentiation compared to controls that were not functionalized with glycosaminoglycan-binding peptides. Hydrogels functionalized with CS-binding peptides also led to significantly higher MMP7 gene expression and activity while the HA-binding peptides significantly increased chondrogenic differentiation of the hMSCs. Our results highlight the potential of this novel biomaterial to modulate cell-mediated processes and create functional tissue engineered constructs for regenerative medicine applications.

## 1 Introduction

Articular cartilage is a complex connective tissue covering the surfaces of bones in synovial joints [1] that enables low-friction articulation and helps transmit and distribute forces to the subchondral bone [2]. It is characterized by a depth-dependent zonal organization and biochemical microenvironment that is vital for its multiple functions [3]. The avascular and aneural nature of articular cartilage contribute to its limited capacity for self-repair following trauma or disease. Current clinical treatments to induce articular cartilage tissue repair, such as autologous chondrocyte implantation, mosaicplasty, and microfracture, often provide short-term pain relief and recovered joint mobility, but the long-term benefits remain elusive. The resulting repaired tissue does not exhibit the same biomechanical behavior as that of native articular cartilage and eventually breaks down, requiring additional treatment such as total joint arthroplasty [1], [3], [4]. As a result, research into the development of bioengineered constructs, with the aim to provide an adequate cellular environment to favor the regeneration of damaged or diseased articular cartilage, has gained momentum in recent years [5], [6], [7], [8], [9], [10]. The ability to engineer tissues that mimic the complex native articular cartilage composition and architecture holds great promise towards restoring the unique biomechanical behavior and function necessary for long-term success.

Strategies for cartilage tissue engineering typically use a combination of biomolecules and biomaterials to provide the appropriate signaling and support for specific cell types [5], [6], [7], [8], [9], [11]. Hydrogels are three-dimensional (3D) water-based matrices that can be engineered to incorporate bioactive cues and be biodegradable [12], [13], thereby providing a pericellular microenvironment reminiscent of the native tissue for encapsulated cells [14]. Poly(ethylene glycol) (PEG) [12], [13], [14], [15], [16] hydrogels have been used extensively for cartilage tissue engineering applications because of their bio-inert nature and versatility. Bioactivity has been introduced into this system through the inclusion of growth factors such as transforming growth factor β3 (TGF-β3) [11] and peptides [10], [17], [18], [19] to markedly enhance the functional outcome. Hydrogels derived from hyaluronic acid (HA) [17] and collagen [18] have also been used for cartilage tissue engineering applications because they are commonly found in native articular cartilage extracellular matrix (ECM). These hydrogels can be degraded *in vivo* but are typically cross-linked with non-degradable cross-linkers that can affect their degradation behavior [12], [14], [15], [17]. Biodegradable hydrogels are an attractive approach for cartilage tissue engineering because they offer a temporary support structure for encapsulated cells but can be degraded in conjunction with the deposition of neo-cartilaginous matrix [15], [20], [21]. Ideally, a hydrogel would degrade at a rate that allows it to provide adequate support to cells without restricting the matrix deposition and remodeling [17].

The incorporation of hydrolytically or enzymatically cleavable substrates are the most common approaches used to impart biodegradability within hydrogel systems [20]. A major drawback of hydrolytically degradable hydrogels is the lack of control over degradation kinetics since the degradation mechanism is not specific. Enzymatically cleavable sites take advantage of cell-mediated processes that can naturally break down the construct, allowing for improved spatiotemporal control of cell migration, degradation, and matrix deposition. Specific enzyme-sensitive peptides, for example, can be included in the hydrogel network to enable localized cell-induced degradation. Matrix metalloproteinases (MMPs) are often targeted as the route of enzymatic degradation since they are known to be involved in the cleavage of ECM components during native tissue remodeling [13], [15], [20], [22], [23], [24], [25], [26]. In articular cartilage, MMPs are involved in the turnover of several matrix components including collagen type II and aggrecan, which aid in cell tissue development and remodeling [13], [15]. MMP7 is thought to play a role in chondrogenesis by controlling the bioavailability of chondrogenic factors and facilitating collagen type II maturation [20]. Recently, MMP7-cleavable peptide substrates (MMP7) were developed and incorporated into PEG hydrogels and shown to degrade via MMP7 secreted by encapsulated human mesenchymal stem cells (hMSCs) during chondrogenesis [20].

**Fig. 1:**
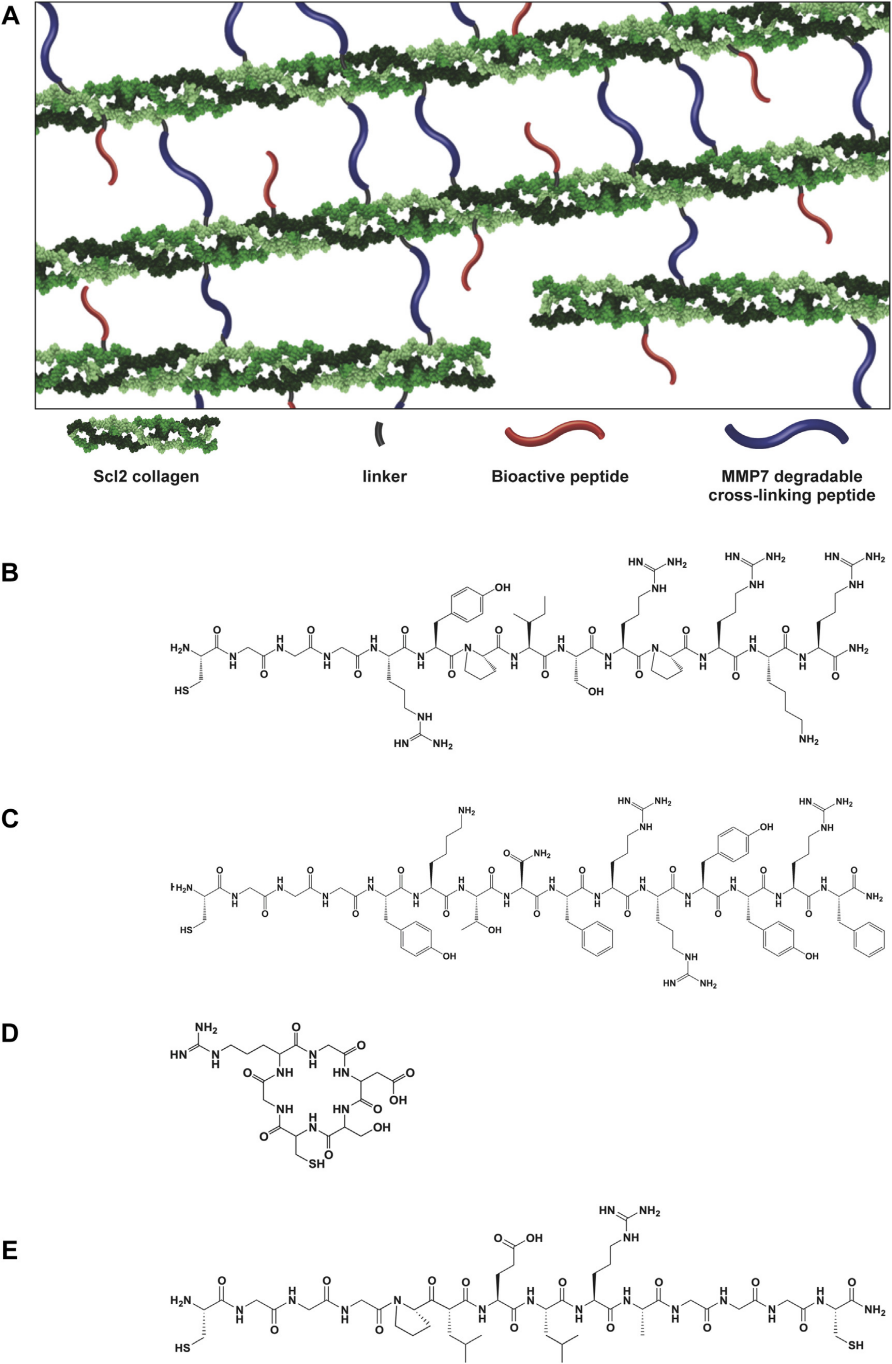
(A) Schematic diagram of peptide-functionalized Scl2 protein hydrogels. The terminal ε-amines of lysines on the Scl2 proteins were modified with a heterobifunctional linker using NHS-ε-amine conjugation chemistry to generate acrylate-functionalized Scl2 proteins. The peptides (B) HAbind, (C) CSbind, (D) RGDS and (E) MMP7 were conjugated to the acrylated Scl2 proteins via thiol-acrylate reactions.

Here, we have developed MMP7-degradable hydrogels based on recombinant Streptococcal collagen-like 2 (Scl2) proteins and functionalized with glycosaminoglycan (GAG)-binding peptides. Scl2 proteins contain the characteristic repeating (Gly-Xaa-Yaa)_n_ residues that assemble into the triple helical conformation found in mammalian collagens. However, unlike mammalian collagens, these proteins inherently lack cell-binding sites and thereby provide a structurally sound biological blank slate by which to systematically integrate specific motifs for a desired cellular response [27], [28], [29], [30], [31], [32]. In addition, Scl2 proteins are produced recombinantly with minimal batch-to-batch variation in predictability of performance, purity, and quality [27], [28], [29], [30], [31]. Previously, Scl2 proteins with integrin-binding sequences have been used in PEG-based hydrogels to bind endothelial and smooth muscle cells for vascular grafts [29]. Here, a blank slate Scl2 protein was cross-linked with the MMP7-sensitive peptide to form hydrogels and functionalized with peptides that bind hyaluronic acid (HA) and chondroitin sulfate (CS) (Fig. 1). Our group recently showed that the HA-binding (HAbind) and CS-binding (CSbind) peptides can specifically and dynamically bind HA and CS, respectively, mimicking native ECM-like interactions [10]. In the current work, we investigated how GAG-binding peptides affect the chondrogenic differentiation of hMSCs encapsulated within the hydrogels that could then be implanted to treat focal defects, MMP7 gene expression and activity. These hydrogels also offer the potential for a less invasive, injectable approach to stimulate chondrogenesis in host MSCs. Moreover, these versatile hydrogels have the potential to incorporate multiple and different peptides to specifically tune bioactivity and biodegradability, that can be tethered from the protein backbone to mimic the complexity of native ECM.

## 2 Materials and methods

### 2.1 Materials

All chemicals were used as provided by the manufacturers. Poly(ethylene glycol)-acrylate-*N*-hydroxysuccinimide (2000 g/mol) (PEG-acrylate-NHS) was purchased from JenKem Technology (Allen, Texas, USA). Rink amide resin, Fmoc-protected amino acids, *N*, *N* dimethyl formamide (DMF), dichloromethane (DCM), 20% (v/v) piperidine in DMF, O-benzotriazole-*N*,*N*,*N'N'*-tetramethyluronium-hexafluoro-phosphate (HBTU), and diisopropylethylamine (DIEA) were purchased from AGTC Bioproducts (Hessle, UK). MMP7 fluorogenic substrate was purchased from Merck Millipore (Nottingham, UK). All other chemicals were purchased from Sigma–Aldrich (Milwaukee, WI, USA). Recombinant Scl2 protein was expressed in *Escherichia coli* BL21-DE3 and purified as previously described [30]. Recombinant Scl2 protein was dialyzed against phosphate-buffered saline (PBS), pH 7.4, and protein concentration was determined using a calculated extinction coefficient.

### 2.2 Peptide synthesis and purification

The HAbind (CGGGRYPISRPRKR), CSbind (CGGGYKTNFRRYYRF), MMP7 (CGGGPLELRAGGGC), and scrambled MMP7 (ScrMMP7; CGGGPALLREGGGC) peptides were synthesized manually on a 2 mmol scale using standard Fmoc solid phase peptide synthesis techniques as previously described [10]. For each coupling, the Fmoc protecting group was removed with 20% (v/v) piperidine in DMF followed by washing with DCM and DMF. Amino acids were activated by adding 4 molar equivalent of each Fmoc protected amino acid to 3.95 molar equivalent of HBTU and dissolved in DMF. Six molar equivalent of DIEA was added to the amino acid solution and the coupling solution added to the resin. The coupling reaction was allowed to proceed for two to three hours before the resin was washed in DCM and DMF. Ninhydrin tests were performed after each Fmoc deprotection and coupling step to monitor the presence of free amines. Once the synthesis was completed, the peptides were cleaved in 95% (v/v) trifluoroacetic acid (TFA), 2.5% (v/v) triisopropyl silane (TIS), and 2.5% (v/v) H_2_O for four hours. TFA was removed using rotary evaporation, and the peptide residue was precipitated and washed with cold diethyl ether (DEE) by centrifugation. The peptide precipitate was then allowed to dry under vacuum to remove residual ether. The peptide was purified (Fig. S1) using reversed phase preparative high performance liquid chromatography (HPLC; Shimadzu) in an acetonitrile/water gradient under acidic conditions on a Phenomenex C18 Gemini NX column (5 μm pore size, 110 Å particle size, 150 × 21.2 mm). Following purification, the peptide was lyophilized on a freeze dryer (Labconco) for storage prior to use. The purified peptide mass was verified by matrix assisted laser desorption spectroscopy (MALDI; Waters).

A cyclic RGDS peptide (GRGDSC) was synthesized at a 1 mmol scale on a 2-chlorotrityl chloride resin (100–200 mesh; VWR). Fmoc-Cys(Trt)-OH (Novabiochem) was dissolved at 1 molar equivalent in DCM with a small amount of DMF added until fully solubilized. Half the solution was added to the resin along with 500 μL of DIEA for 15 min shaking on a wrist action shaker. This was repeated with the remaining solution followed by thorough washing with DMF and DCM. A ninhydrin test was performed to monitor the coupling by detecting the presence of free amines. The remaining free amines were capped by adding a solution of 5% (v/v) acetic anhydride (Sigma) with 2.5% (v/v) DIEA in DMF for 10 min with shaking, and repeated with 5 min shaking using fresh solution. The resin was then washed thoroughly with DCM and DMF before the ninhydrin test. The Fmoc protecting group was removed as described above and Fmoc-Asp(Otbu)-Ser(psiMe, Mepro)-OH (Merck) was coupled at 2 molar equivalents with 1.95 molar equivalents of HBTU and 3 molar equivalents of DIEA in DMF. The remaining free amines were capped, and all other amino acids were coupled as described above. The protected peptide was cleaved from the resin by adding 10 mL of 5% (v/v) TFA in DCM for 10 min with shaking. The solution was drained into a round bottom flask and the resin rinsed with DCM until the solution in the synthesis vessel was clear. The DCM and TFA were removed carefully by rotary evaporation, leaving approximately 40 mL of solution to avoid cleaving the protecting groups from the peptide. Ammonium hydroxide (10 mL) was added to neutralize the TFA followed by acetonitrile to increase peptide solubility. The protected peptide (Fig. S2 and S3) was purified by reversed phase preparative HPLC running a mobile phase gradient of 80% ultrapure H_2_O and 20% (v/v) ACN to 100% (v/v) ACN with 0.1% (v/v) TFA. The solvent was removed by rotary evaporation until the protected peptide was completely dry, and then re-dissolved in DMF at 1 mg/mL. The peptide was cyclized by adding 2 equivalents of benzotriazol-1-yl-oxytripyrrolidinophosphonium hexafluorophosphate (PyBop; AGTC Bioproducts) and 3 equivalents of DIEA overnight. The DMF was removed by rotary evaporation and the remaining product was re-dissolved in acetonitrile/water until solubilized and purified by HPLC as described above. The remaining protecting groups were removed using 95% (v/v) TFA with 2.5% (v/v) H_2_O and 2.5% (v/v) TIS. The peptide was precipitated in cold DEE and purified by HPLC. Liquid chromatography-mass spectrometry (LC-MS) was performed on an Agilent 6130 Quadrupole LC-MS coupled to an Agilent 1260 Infinity LC using a 150 × 4.6 mm Phenomenex Gemini NX C18 column with a 5 μm pore size and 100 Å particle size. Ultrapure H_2_O and acetonitrile each containing 0.1% (v/v) formic acid (VWR) by volume were used for the mobile phase at a flow rate of 1 mL/min. The peptide was eluted with a gradient of 95% (v/v) H_2_O to 95% (v/v) acetonitrile over 11 min. The electrospray source was operated with a capillary voltage of 3.2 kV and a cone voltage of 25 V with nitrogen used as the nebulizer and desolvation gas at a total flow of 600 L/h. Although cyclic GRGDSC did not stick to the column, electrospray ionization (ESI) of an early elution confirmed the correct mass.

### 2.3 Streptoccocal collagen-like 2 (Scl2) protein synthesis and purification

Blank slate bacterial (non-animal) collagen-like protein was obtained using the Cold-shock vector (Takara Bio, Shiga, Japan) [33] for expression of pColdIII-V-CL in *E. coli* following methods previously described [34]. Briefly, the construct was derived using pColdIII-163 encoding p163 polypeptide based on Scl 2.28 [35], [36]. A His_6_-tag sequence was introduced at the N-terminal of the p163 polypeptide sequence and a protease cleavage LVPRGSP sequence was inserted between the N-terminal globular domain (V) and the following collagen-like domain (CL) sequences [34]. The pColdIII-VCL construct was expressed in *E. coli* BL21-DE3 strain. Cells were grown in 2 x YT media with ampicillin (100 μg/mL) at 37 °C, shaking at 200 rpm until A600 reached an optical density (OD) in the range 3–6 AU. Cells were then cooled to 25 °C and 1 mm isopropyl beta-d-thiogalactopyranoside (IPTG) was added to induce protein expression. After 10 h incubation, cells were further cooled to 15 °C for 14 h after which the cells were harvested by centrifugation. For the extraction of protein, each 1 g of cell paste was re-suspended in 20 mL of 50 mm acetic acid at pH 2, and the cells were ruptured by sonication using a Misonix S4000 instrument with a Enhance Booster #1 probe at 30 A (instrument scale) for 30 min. The cell debris was removed by centrifugation at 4 °C and the supernatant containing the V-CL was retained. The supernatant was kept at 4 °C for 16 h and any precipitate that had formed was removed by centrifugation. The collagen-like domain was obtained from purified V-CL by incubating with 0.01 mg/mL protease at 4 °C for 24 h to remove the V-domain. The pH of the supernatant was then adjusted to 8 using 50 mm Na_2_HPO_4_ and 1 m NaOH. The purified collagen-like domain was then concentrated and had its buffer exchanged using a 10 kDa cross-flow filtration membrane (Pall Sciences). Purity was verified by sodium dodecyl sulfate-poly-acrylamide gel electrophoresis (SDS-PAGE) [34], [37] and MALDI mass spectrometry.

### 2.4 Scl2 modification with acrylate groups

The Scl2 protein contains ∼8.6 mol% lysine groups on its backbone that can be functionalized with a heterobifunctional linker using a method previously reported [29]. Briefly, Scl2 protein was dispersed at 100 mg/mL in 50 mm acetic acid in H_2_O at room temperature, and the pH was adjusted to 8.5 using 1 m NaOH. Approximately 33.3% of the terminal amines on the available lysines were reacted with PEG-acrylate-NHS to generate Scl2 protein with acrylate groups (acrylate-Scl2). The reaction was allowed to proceed for 18 h at room temperature with agitation. Excess PEG-acrylate-NHS and any reaction by-products were removed by dialysis against H_2_O overnight. The acrylate-Scl2 samples were sterile-filtered, lyophilized, and stored at 4 °C prior to use.

### 2.5 Scl2 conjugation to GAG-binding peptides

HAbind, CSbind and RGDS peptides were conjugated to acrylate-Scl2 via thiol-acrylate chemistry [38]. Briefly, acrylate-Scl2 protein was re-suspended at 100 mg/mL in 50 mm acetic acid in H_2_O at room temperature. The pH was adjusted to 8.5 using 1 m NaOH. The HAbind and CSbind peptides were added to modify 5 or 10% of the acrylates. The RGDS peptide was reacted to modify 10% of the acrylates. The reaction was allowed to proceed for 2 h at room temperature with agitation. Unconjugated peptide and other reaction by-products were removed via dialysis against H_2_O overnight. The functionalized Scl2 proteins (HAbind-Scl2, CSbind-Scl2, RGDS-Scl2) were sterile-filtered, lyophilized, and stored at 4 °C until needed.

### 2.6 Characterization of functionalized Scl2 proteins

HAbind-Scl2, CSbind-Scl2, and RGDS-Scl2 were characterized using Fourier transform infrared spectroscopy (FTIR) and cross-polarization magic-angle spinning (CP-MAS) solid-state ^13^C NMR to determine conjugation efficiency. FTIR analysis with a Perkin Elmer Spectrum One spectrometer was used to evaluate the conjugation of the linker and tethered peptides to Scl2 protein as previously described [29]. FTIR spectra were taken with a scanning wavenumber range from 4000 to 650 cm^−^^1^. CP-MAS solid-state ^13^C NMR analysis experiments were performed using a Bruker Avance III 200 MHz (4.7T) spectrometer at a Larmor frequency of 50.1 MHz and spinning speed of 8 kHz in a 4 mm rotor using a contact time of 1 ms. The 1.5 ppm peak of tetrakis(trimethylsilyl)methane was used for reference. Data were processed with 200 Hz line broadening.

Circular dichroism (CD) spectra of functionalized Scl2 proteins in H_2_O were recorded on a Jasco J-715 spectropolarimeter controlled by the Jasco Spectra Manager software equipped with a Jasco PTC-348WI Peltier temperature control system using a quartz cuvette with a path length of 0.1 or 1 mm. For determining the thermal transitions, the ellipticity at 220 nm was monitored [30] as the sample temperature was increased from 25 to 45 °C, with an average temperature slope of 10 °C/h. The ellipticity was normalized to path length and number of residues and was plotted against temperature.

### 2.7 Preparation of Scl2 hydrogels

To generate hydrogels, acrylate-Scl2, HAbind-Scl2, CSbind-Scl2 or RGDS-Scl2 samples were re-suspended at 100 mg/mL in chondrogenic medium (medium defined in Section 2.10) at room temperature with the pH adjusted to 8.5 using 1 m NaOH. The MMP7 or ScrMMP7 peptide was dissolved in 4 mm triethanolamine (TEA) in chondrogenic medium at pH 8.5 and reacted to modify 90% of the acrylates. The resulting solutions were sterile-filtered and pipetted to generate homogeneous formation of MMP7-Scl2, HAbind-MMP7-Scl2, CSbind-MMP7-Scl2 or RGDS-MMP7-Scl2 hydrogels.

### 2.8 Characterization of Scl2 hydrogels

Hydrogels were characterized using CP-MAS solid-state ^13^C NMR, rheology, and compression testing. CP-MAS solid-state ^13^C NMR analysis was used to quantify the conjugation of MMP7, HAbind, CSbind, and RGDS peptides to Scl2 protein as previously described. The mechanical properties of the hydrogels were studied using oscillatory shear rheology and confined compression testing. Oscillatory parallel plate rheological measurements were performed using an Advanced Rheometer AR2000ex with AR Instrument Software (TA instruments) fitted with a Peltier temperature control system. Samples were tested at 37 °C using an 8 mm diameter parallel steel plate. All samples were individually prepared immediately prior to testing. Three sequential sweeps were applied; (1) time sweep for 2 h at 0.1% strain and 6.28 rad/s angular frequency, (2) angular frequency sweep from 0.01 to 100 rad/s at 0.1% strain and (3) strain sweep from 0.01 to 100% at 6.28 rad/s angular frequency. For all samples, a compression load of 0.5 N was exerted during testing. For all compression tests, discs 8 mm in diameter and 2 mm in thickness were prepared from hydrogels swollen in chondrogenic medium for at least 24 h and inserted in a confined ring; dimensions were measured using digital calipers. Swollen hydrogels were mechanically tested in compression using an Instron Model 5540 testing machine (Norwood, MA, USA) equipped with a 50 N load cell. Samples were pre-loaded to 0.05 N and compressed to 10% strain at a crosshead speed of 0.5% strain/min. Compressive modulus was calculated from the linear portion of the stress–strain curve.

### 2.9 hMSC culture

Bone marrow-derived hMSCs were purchased from PromoCell GmbH (Heidelberg). The hMSCs were seeded at 4000 cells per cm^2^ in T225 flasks and cultured in mesenchymal stem cell growth medium (MSCGM) (PromoCell GmbH, Heidelberg). hMSCs were incubated at 37 °C and 5% CO_2_ and the medium changed every three days. The cells were harvested at 80% confluency with 0.025% (w/v) trypsin-EDTA (Sigma Aldrich, UK) in PBS, centrifuged and subcultured in MSCGM. Passage 6 hMSCs were used for all hydrogel encapsulation experiments.

### 2.10 Cell encapsulation and culture

hMSCs were homogeneously dispersed at 8 × 10^6^ cells per mL in the pre-made 100 mg/mL acrylate-Scl2, HAbind-Scl2, CSbind-Scl2 or RGDS-Scl2 solution containing chondrogenic medium (high-glucose (4.5 g/L) Dulbecco's Modified Eagle Medium (DMEM; Invitrogen) supplemented with 0.1 mm dexamethasone (Sigma Aldrich, UK), 1% (v/v) penicillin streptomycin (Sigma Aldrich, UK), 50 μg/mL l-proline (Sigma Aldrich, UK), 50 μg/mL ascorbate-2-phosphate (Sigma Aldrich, UK), 1 x insulin-transferrin-selenium (ITS) Premix (BD Biosciences, Oxford, UK), and 10 ng/mL TGF-β3 (Lonza, Slough, UK)). The MMP7 peptide was prepared in 4 mm TEA in chondrogenic medium and mixed with the protein/cell solution. Aliquots (50 μL) of the resulting mixture were injected in a non-tissue culture treated 48-well plate and allowed to gel. The hydrogels were then cultured in 1 mL of chondrogenic medium. Hydrogels were incubated at 37 °C and 5% CO_2_ for four weeks with the medium changed every three days.

### 2.11 Degradation testing and MMP7 activity assay

Degradation of the hydrogels was determined by freeze-drying the hydrogels and measuring the dry weight change over time, with and without encapsulated cells. Hydrogels were prepared as previously described and incubated in chondrogenic medium for 24 h at 37 °C and 5% CO_2_. Cell-free hydrogels were incubated in chondrogenic medium with exogenous MMPs (30 ng/mL) at 37 °C and 5% CO_2_ for one week with medium changed daily and dry weight measurements taken at each day. Percentage weight change was normalized to day 0. Degradation by recombinant human MMP1, MMP2, MMP7, and MMP13 (AnaSpec, San Jose, CA, USA) was tested against negative control (chondrogenic medium alone) and a positive control (0.2 μg/mL trypsin).

Cell-seeded hydrogels were incubated in chondrogenic medium at 37 °C and 5% CO_2_ for four weeks, and dry weight measurements were taken after 0, 1, 3, 7, 14, 21, and 28 days of culture. Percentage weight change corresponding to the cumulative effect of cell proliferation, cartilage-like matrix deposition, and hydrogel degradation was normalized to day 0. At each time point, 1 mL of medium was also removed, sterile-filtered, and analyzed for MMP7 activity. MMP7 activity was determined using a fluorogenic MMP7 substrate assay (Merck Millipore, Nottingham, UK) and compared to negative control (chondrogenic medium) and positive control (recombinant human MMP7) according to the manufacturers' instructions.

### 2.12 Cell viability

hMSC-seeded hydrogels were cultured for 0, 1, 3, 7, 14, 21, and 28 days. After the culture period, the hydrogels were washed three times in PBS and analyzed for cell viability. The viability of cells was qualitatively assessed with a LIVE/DEAD^®^ Viability/Cytotoxicity Kit (Molecular Probes, Inc., Eugene, OR) according to the manufacturers' instructions. Fluorescence confocal microscopy (Leica SP5 inverted microscope, Leica Microsystems, UK) was used to visualize live (calcein; green) and dead (ethidium homodimer-1; red) cells. The viability of cells in the hydrogels was also quantified by the CellTiter-Glo^®^ assay (Promega). This assay is based on the luminescent signal output produced by metabolically active cells based on the level of ATP present. All data were normalized to DNA content present at each time point.

### 2.13 DNA, sGAG, and hydroxyproline quantification

hMSC-seeded hydrogels were cultured for 0, 1, 3, 7, 14, 21, and 28 days. After the culture period, they were washed three times in PBS and digested individually in papain digest solution (2.5 units papain/mL, 5 mm cysteine HCl, 5 mm EDTA, in PBS (all reagents from Sigma Aldrich)) at 60 °C for 24 h. Papain digests were stored at −20 °C until further analysis. Digested samples were assayed for total DNA content using the Quant-iT™ PicoGreen^®^ Kit (Invitrogen) according to the manufacturers' instructions. The standard curve was generated with dsDNA (Invitrogen).

Sulfated GAG (sGAG) content was quantified using the Blyscan Kit (Biocolor, Carrickfergus, UK) according to the manufacturers' instructions. The standard curve was generated with bovine trachea chondroitin sulfate A (Sigma–Aldrich).

Total collagen content was determined using the hydroxyproline content as a measure of total collagen. Unlike mammalian collagens, bacterial collagens lack hydroxyproline, which enabled us to distinguish between the collagen in the hydrogel and new collagen formation. Digested samples were hydrolyzed in 6 N HCl at 110 °C for 18 h. The hydroxyproline content of the hydrolysate was determined using the chloramine-T/Ehrlich's reagent assay and the color change quantified spectrophotometrically at 560 nm [20], [39]. Briefly, free hydroxyproline was oxidized using chloramine-T, resulting in the formation of pyrrole while Ehrlich's reagent resulted in the formation of the chromophore. The standard curve was generated with l-hydroxyproline (Sigma Chemical Co.).

### 2.14 Histology and immunohistochemistry of hydrogels

After 0 and 28 days of culture, hMSC-seeded hydrogels were washed three times in PBS, fixed with 4% (v/v) paraformaldehyde (Electron Microscopy Sciences, PA, USA) for 1 h at 4 °C, washed three times in PBS, permeabilized with 0.4% (v/v) Triton X-100 (Sigma Aldrich, UK) for 1 h and washed again. Hydrogels were flash frozen in OCT (Tissue-Tek, Fisher Scientific) and cryosectioned at a thickness of 10 μm. Sections were transferred to treated slides (Superfrost Plus, Thermo Scientific) and allowed to adhere for 24 h at 4 °C. Slides were stained for deposited sGAG with alcian blue (pH 2.5), and for cell nuclei and matrix with haematoxylin and eosin (H&E).

Immunohistochemical staining (IHC) was performed for collagen type I (Abcam), collagen type II (Abcam), and collagen type X (Abcam) with rabbit IgG and PBS negative controls. Samples were pre-treated with hydrogen peroxide, an avidin and biotin blocking kit (Vector Labs, UK), and blocked with 5% (v/v) goat serum. Primary antibodies were incubated overnight at 1/200 in 5% (v/v) goat serum, followed by goat-anti-rabbit secondary antibody labeled with HRP at 1:100 for 1 h, stained with a 3,3′-diaminobenzidine (DAB) kit (Vector Labs, UK) for 10 min, and counter-stained with haematoxylin. All stained sections were dehydrated, mounted with Histomount (Fisher Scientific, UK), and viewed on an Olympus BX51 microscope equipped with an Olympus DP70 camera.

### 2.15 Gene expression analysis

hMSC-seeded hydrogels were cultured for 0, 1, 3, 7, 14, 21, and 28 days. After the culture period, they were washed three times in PBS. Total RNA was isolated using a tissue ruptor (Qiagen) to homogenize samples with RLT buffer after which QIAshredder columns (Qiagen) and the RNeasy Mini Kit (Qiagen) were used to extract the RNA according to the manufacturers′ instructions. QuantiTect^®^ Reverse Transcription Kit (Qiagen) and QuantiTect^®^ SYBR Green PCR Kit (Qiagen) were used to perform reverse transcription and quantitative real-time polymerase chain reaction (qPCR), respectively. Thermocycling and SYBR Green detection were performed on a Corbett Rotorgene 6000 (Qiagen) with extension at 72 °C and denaturing at 95 °C. Annealing temperatures varied depending on the primers. Data were analyzed using the ΔΔCT method [40]. The following primers were used: MMP7 (Forward 5′-GAGTGAGCTACAGTGGGAACA-3′ and Reverse 5′-CTATGACGCGGGAGTTTAACAT-3′), and TIMP2 (Forward 5′-TGGACGTTGGAGGAAAGAAG-3′ and Reverse 5′-GGGCACAATGAAGTCACAGA-3′) at an annealing temperature of 52 °C, GAPDH (Quiagen) (Forward 5′-TGGTATCGTGGAAGGACTCATGA-3′ and Reverse 5′-ATGCCAGTGAGCTTCCCGTTCAG-3′), COL1A1 (Forward 5′-CATTAGGGGTCACAATGGTC-3′ and Reverse 5′-TGGAGTTCCATTTTCACCAG-3′), COL2A1 (Forward 5′-CATCCCACCCTCTCACAGTT-3′ and Reverse 5′-GTCTCTGCCTTGACCCAAAG-3′), and ACAN (Forward 5′-CACTGTTACCGCCACTTCCC-3′ and Reverse 5′- GACATCGTTCCACTCGCCCT -3′) at an annealing temperature of 60 °C, and SOX9 (Forward 5′-AACGCCGAGCTCAGCAAG-3′ and Reverse 5′- ACGAACGGCCGCTTCTC-3′) at an annealing temperature of 62 °C.

### 2.16 Statistical analysis

All experimental groups had a sample size of n = 3 for biochemical, qPCR, and mechanical property analyses. All cell-related work was repeated with hMSCs from three different donors and with each donor having a sample size of n = 3. Data are presented as means ± standard deviation (SD). Statistical significance was determined by performing analysis of variance (ANOVA) with a significance accepted at *p*-value < 0.05, except for gene expression analysis where ANOVA with Bonferroni correction was performed.

## 3 Results and discussion

### 3.1 Scl2 functionalization

FTIR spectroscopy was used to analyze and validate each functionalization step. It showed IR transmittance peaks at 1630 cm^−^^1^ (amide C=O) that were assigned to the Scl2 protein in all samples and used for normalizing. The peak at 1110 cm^−^^1^ (ether C–O–C) assigned to the acrylate was present in the acrylate-Scl2, HAbind(10%)-Scl2, CSbind(10%)-Scl2, and RGDS(10%)-Scl2 samples and absent in the Scl2 control (Fig. S5). CP-MAS solid-state ^13^C NMR was used to quantify the conjugation density of acrylate, MMP7, HAbind, CSbind, and RGDS peptides to Scl2 (Table S1 and Fig. S6). CP-MAS analysis showed the conjugation density of acrylate and each peptide to be at the expected value.

**Fig. 2:**
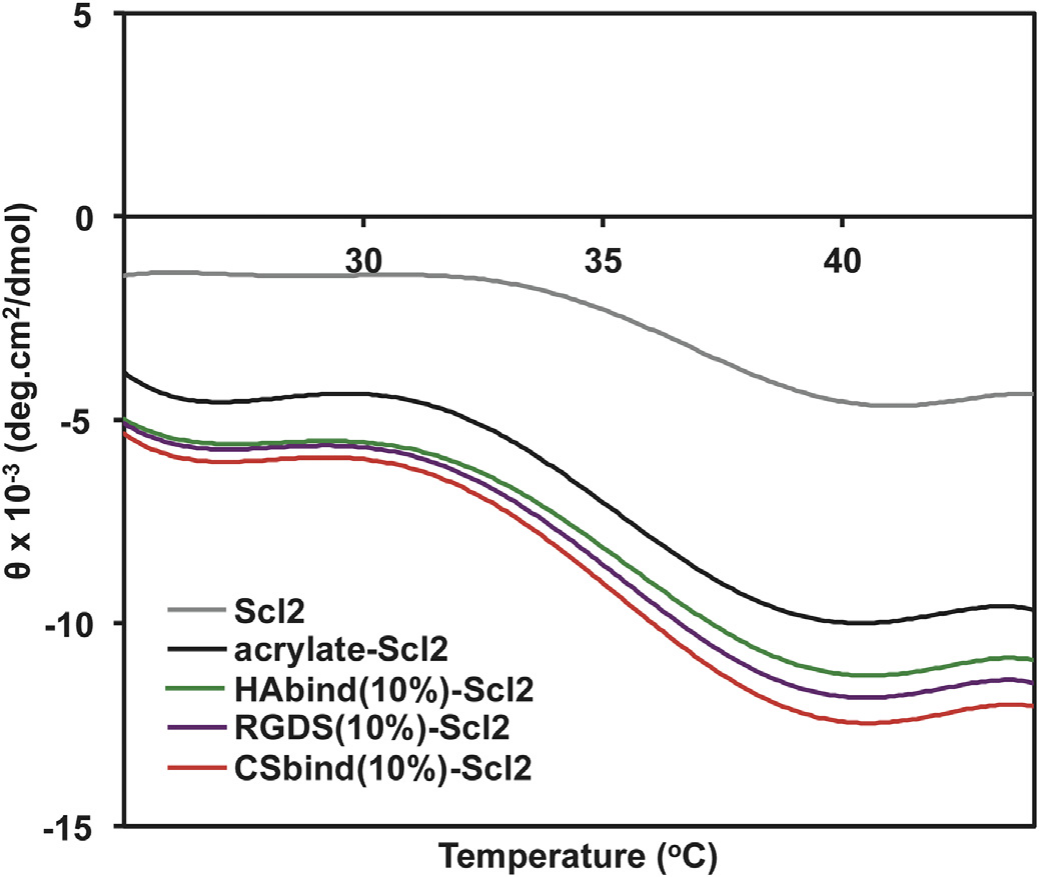
The ellipticity of functionalized Scl2 proteins monitored at 220 nm from 25 to 45 °C. Representative spectra are shown for all samples (n = 3).

CD was used to observe any changes in the secondary structure of the modified proteins as a result of the functionalization. Scl2 proteins typically form a triple helical conformation with a characteristic peak at 220 nm in the CD spectra [29], [34]. The addition of the acrylate and tethered HAbind, CSbind, and RGDS peptides did not affect the triple helical conformation, and the thermal stabilities (Fig. 2) remained essentially unchanged. Formation of a hydrogel by addition of the MMP7 cross-linking peptide made CD determination difficult, but cross-linking is generally seen as stabilizing constructs [30].

### 3.2 Mechanical characterization of hydrogels

**Fig. 3:**
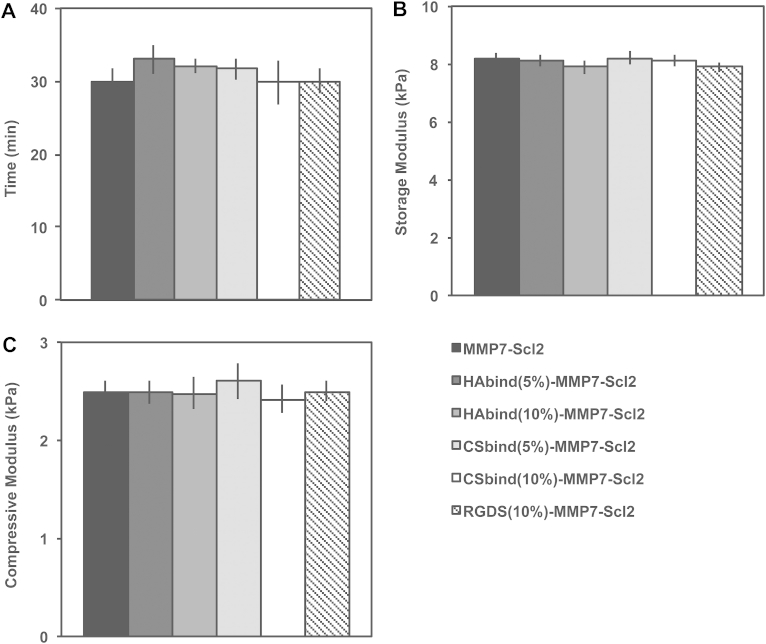
Mechanical properties of functionalized Scl2 hydrogels. (A) Time to gelation at a temperature of 37 °C, angular frequency of 6.28 rad/s and strain of 0.5%. (B) Storage modulus determined from strain sweep up to 1% strain at a temperature of 37 °C and an angular frequency of 6.28 rad/s. (C) Confined elastic modulus of compression of hydrogels compressed to 10% strain at 0.5% strain/min. Values are represented as means ± SD. *P < 0.05 (n = 3).

For all hydrogels, the cross-linking density was kept constant to maintain similar mechanical properties since matrix stiffness has been shown to affect cell processes such as adhesion, proliferation, and differentiation [41], [42]. Oscillatory shear rheology confirmed gelation and was used to measure the mechanical properties of all formulations. There were no statistical differences in time to gelation or storage moduli between the different hydrogel formulations (Fig. 3A, B and Fig. S7). The equilibrium storage moduli of all hydrogels remained in the linear elastic region (∼8 kPa) up to strains of 1% with minimal variation and significantly decreased above 10% strain. These hydrogels appear to retain sufficient mechanical properties up to 10% strain, which corresponds with physiologically relevant values [2], [3], [4], [39]. The failure strains (20–30%) of the hydrogels are comparable to other hydrogel-based materials for treating focal articular cartilage defects [39], [43]. Mechanical behavior of the hydrogels was further characterized using confined compression testing (Fig. 3C and Fig. S8). All hydrogel formulations had similar compressive moduli of ∼2.5 kPa. Since the hydrogels had similar mechanical properties, we were able to compare the influence of the HAbind, CSbind, and RGDS peptides decoupled from the mechanical properties.

### 3.3 Degradation kinetics of acellular hydrogels

**Fig. 4:**
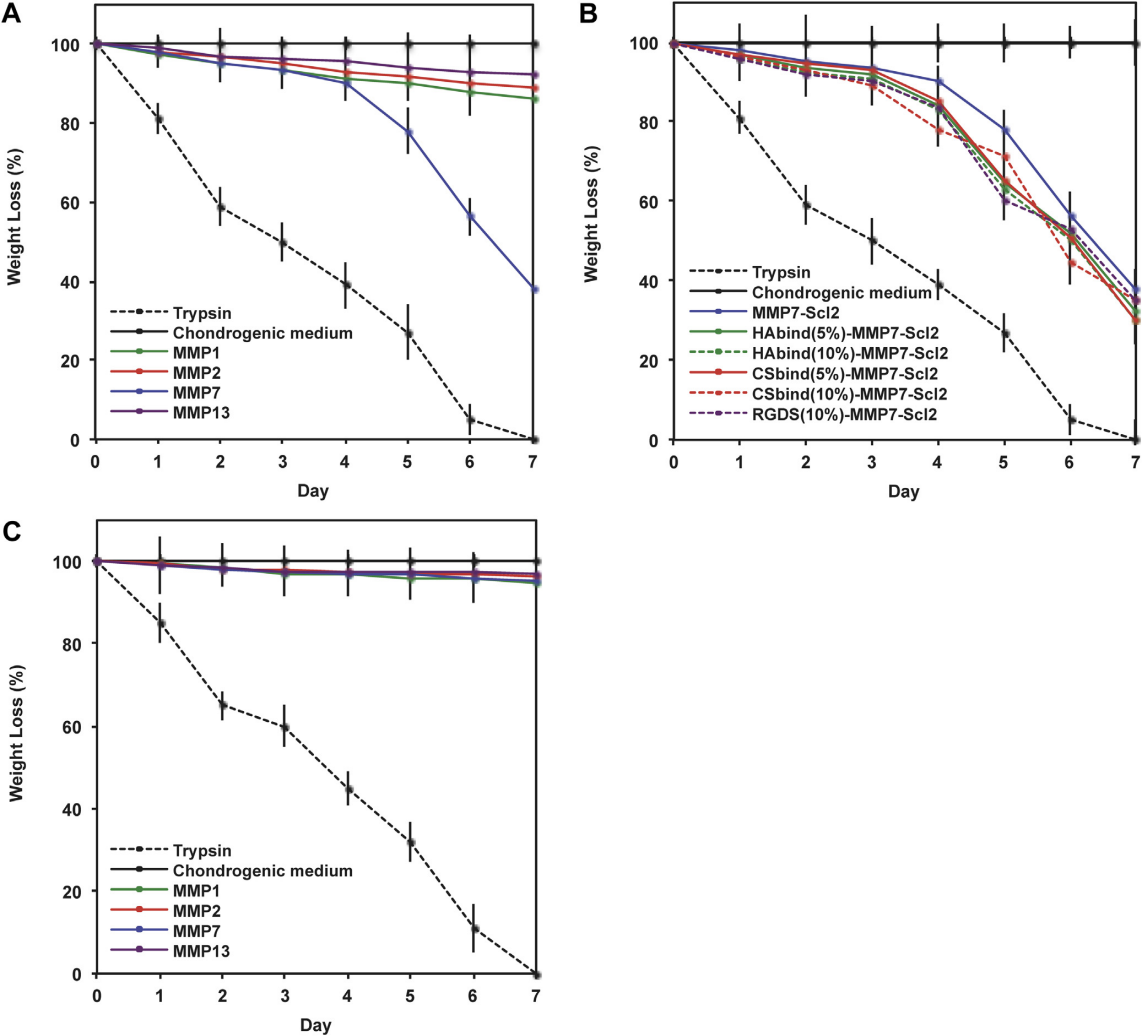
Degradation of acellular hydrogels over time characterized by dry weight loss. Degradation of (A) MMP7 peptide cross-linked-hydrogels without tethered bioactive sequences, (B) MMP7 peptide cross-linked hydrogels with tethered bioactive sequences, and (C) ScrMMP7 peptide cross-linked-hydrogels without tethered bioactive sequences after exposure to exogenous recombinant human MMP1, 2, 7 and 13 in chondrogenic medium. Degradation was normalized to weight at day 0. Trypsin-driven degradation is positive control. Chondrogenic medium without exogenous recombinant human MMP7 is negative control. Values are represented as means ± SD (n = 3).

Fig. 4 illustrates the degradation of the acellular hydrogels in the presence of recombinant human MMP7 compared to trypsin, MMP1, MMP2, and MMP13. As expected, the hydrogels cross-linked with the MMP7-sensitive peptide degraded faster in the presence of MMP7 compared to when exposed to MMP1, MMP2, and MMP13 where minimal degradation was observed (Fig. 4A) (*P* < 0.05). Cleavage of the MMP7 peptide alone by exogenous MMP7 was confirmed through LCMS-ESI analysis (Fig. S4) and suggests that the degradation of the MMP7-Scl2 hydrogels occurs specifically through the MMP7 peptide cross-links being cleaved by MMP7. MMP1, MMP2, and MMP13 caused small amounts of degradation, likely because MMPs are somewhat promiscuous and known to recognize other peptide sequences [13], [20], [45], [46], [47]. The inclusion of bioactive epitopes did not significantly affect the degradation rate in the hydrogels, demonstrating that MMP7 activity is not disrupted by the presence of the bioactive peptides (Fig. 4B).

Control ScrMMP7-Scl2 hydrogels which lack the glutamic acid–leucine cleavage site for MMP7 and have no other recognizable cleavage site for MMP1, MMP2, MMP7 or MMP13 [44], [45], [46], were only slightly degraded by all MMP enzymes (Fig. 4C). All hydrogels were significantly degraded by trypsin, a highly active and efficient enzyme that can cleave more sites in the hydrogels [48], [49], and at faster rates compared to the MMP enzymes tested (*P* < 0.001).

### 3.4 Cell viability

**Fig. 5:**
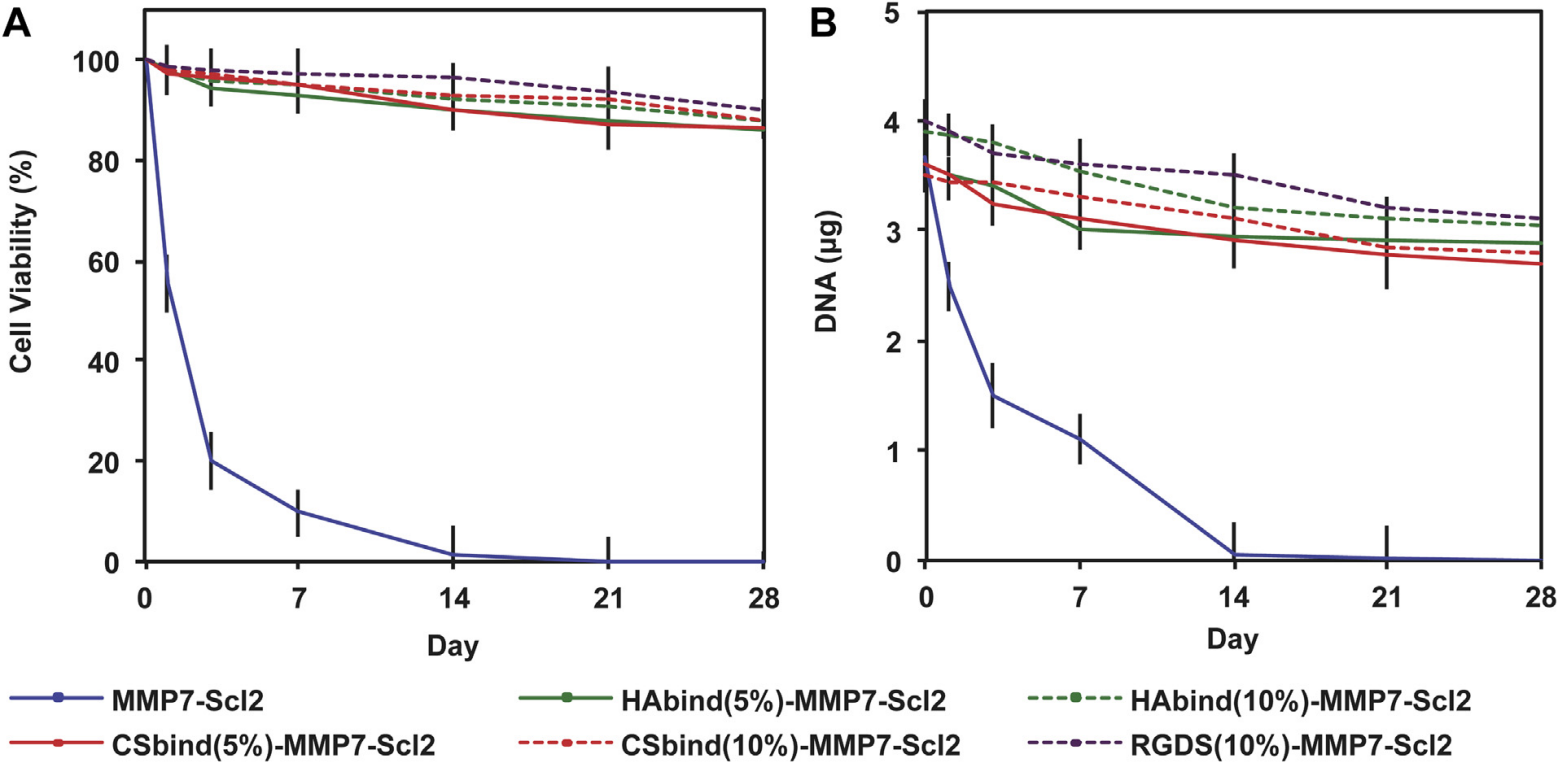
hMSC viability in hydrogels. (A) Cell viability in hydrogels cultured over 4 weeks *in vitro* quantified using the CellTiter-Glo^®^ assay. (B) DNA content per construct in hydrogels cultured over 4 weeks *in vitro* quantified using a PicoGreen^®^ Kit. All data normalized to day 0. *P < 0.05 (n = 3 for each donor, three different bone marrow-derived hMSC donors).

The viability of hMSCs was maintained at significantly higher levels in the HAbind-MMP7-Scl2, CSbind-MMP7-Scl2, and RGDS-MMP7-Scl2 hydrogels compared to the MMP7-Scl2 hydrogels without cell-interactive epitopes (Fig. 5A) (*P* < 0.001). The DNA content correlated with these results showing a significantly higher cell number in hydrogels with bioactive sequences compared to the MMP7-Scl2 control (Fig. 5B) (*P* < 0.001). These differences were expected since the RGDS peptide is known to improve cell adhesion and the HAbind and CSbind peptides are designed to bind HA and CS, which are known to improve cell viability [10], [19], [41]. However, the viability did decrease slightly throughout the culture period for these formulations compared to Day 0. This is likely a result of inefficient diffusion of nutrients, oxygen and waste products during long culture times [42] and may be less of a concern under the cyclic loads present in the joint. The cell viability was further confirmed with confocal imaging of the cells within the gels labeled with the LIVE/DEAD^®^ dyes (Fig. S9).

### 3.5 In vitro chondrogenesis

**Fig. 6:**
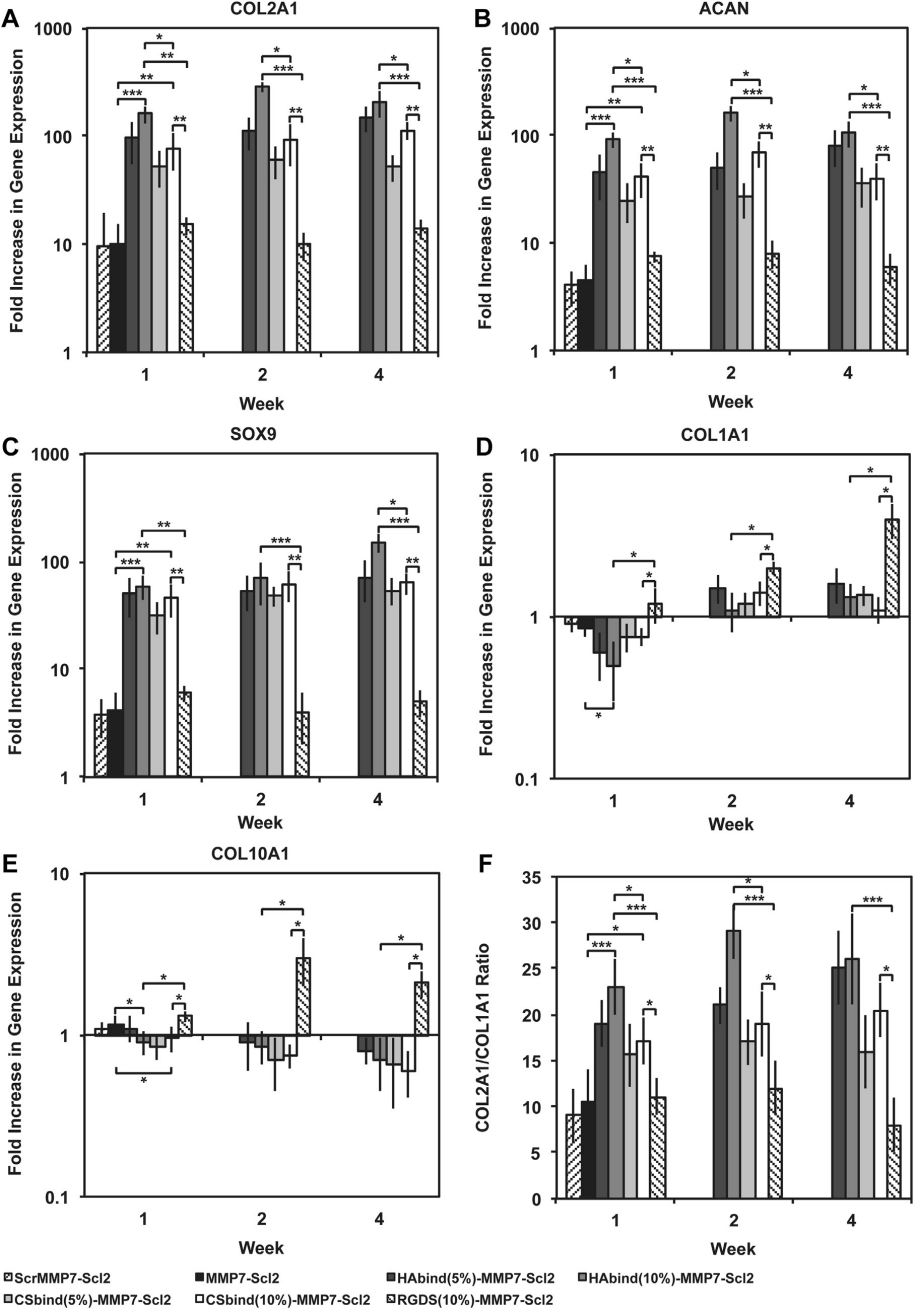
(A) COL2A1, (B) ACAN, (C) SOX9, (D) COL1A1 and (E) COL10A1 gene expression for hMSCs encapsulated in hydrogels over 4 weeks *in vitro*, as measured by qPCR and analyzed using the ΔΔCT method, represented as a fold difference relative to undifferentiated hMSCs (calibrator) prior to encapsulation and normalized to GAPDH. (F) COL2A1/COL1A1 ratio was high throughout the culture period. Values are represented as means ± SD. *P < 0.05, **P < 0.01, ***P < 0.001 (n = 3 for each donor, three different bone marrow-derived hMSC donors).

The effect of the HAbind and CSbind peptides compared to the RGDS peptide was evaluated on the chondrogenic differentiation of encapsulated hMSCs [10], [19], [47]. The gene expression of chondrogenic markers, COL2A1, ACAN, and SOX9 (Fig. 6A, B and C), all indicate the HAbind-MMP7-Scl2 and CSbind-MMP7-Scl2 hydrogels significantly enhanced chondrogenic differentiation of hMSCs. These genes were upregulated at all time points compared to the MMP7-Scl2 and RGDS-MMP7-Scl2 hydrogels (HAbind-MMP7-Scl2, *P* < 0.001; CSbind-MMP7-Scl2, *P* < 0.01). In particular, the higher concentration of HAbind peptide at 10% functionalization correlated with the highest upregulation of COL2A1, ACAN, and SOX9 gene expression compared to all other hydrogels investigated including CSbind-MMP7-Scl2 (*P* < 0.001). The measure of a differentiation index (the ratio of COL2A1/COL1A1 gene expression, Fig. 6F) confirmed the individual gene expression data observed from COL2A1, ACAN, and SOX9 gene expression as it remained significantly higher for the HAbind-MMP7-Scl2 and CSbind-MMP7-Scl2 samples throughout the culture period compared to the MMP7-Scl2 and RGDS-MMP7-Scl2 controls (HAbind-MMP7-Scl2, *P* < 0.001; CSbind-MMP7-Scl2, *P* < 0.05). In contrast to the RGDS samples, this implied maintenance of the chondrogenic gene expression profile during the entire culture period. The HAbind and CSbind peptides showed different extents of chondrogenic differentiation although they are similarly charged and have the potential to interact electrostatically with biomolecules in a similar manner. We previously showed the HAbind and CSbind peptides can be used to spatially organize HA and CS respectively in the same scaffold system, which indicates specific peptide-GAG interactions beyond electrostatic interactions [10], [19]. The presence of the HAbind and CSbind peptides in our hydrogels significantly promoted chondrogenesis, presumably by mimicking protein-GAG interactions in the native microenvironment.

The gene expression levels of COL1A1 and COL10A1 (Fig. 6D and E) were significantly lower for the HAbind-MMP7-Scl2 and CSbind-MMP7-Scl2 hydrogels, indicating inhibited or delayed differentiation of hMSCs towards a hypertrophic phenotype throughout the culture period compared to the RGDS-MMP7-Scl2 and MMP7-Scl2 controls (*P* < 0.05). Limiting the terminal differentiation of hMSCs to a hypertrophic phenotype is a critical challenge toward the clinical use of these cells for articular cartilage regeneration [50]. In the native environment, hypertrophic chondrocytes can induce mineralization of the ECM, leading to inferior function and mechanical behavior of the tissue [50]. Previous studies suggested that the presence of an RGDS peptide sequence may be important during the early stages of chondrogenesis, but its persistence has been shown to hinder the differentiation of hMSCs and the extent of chondrogenesis [13].

**Fig. 7:**
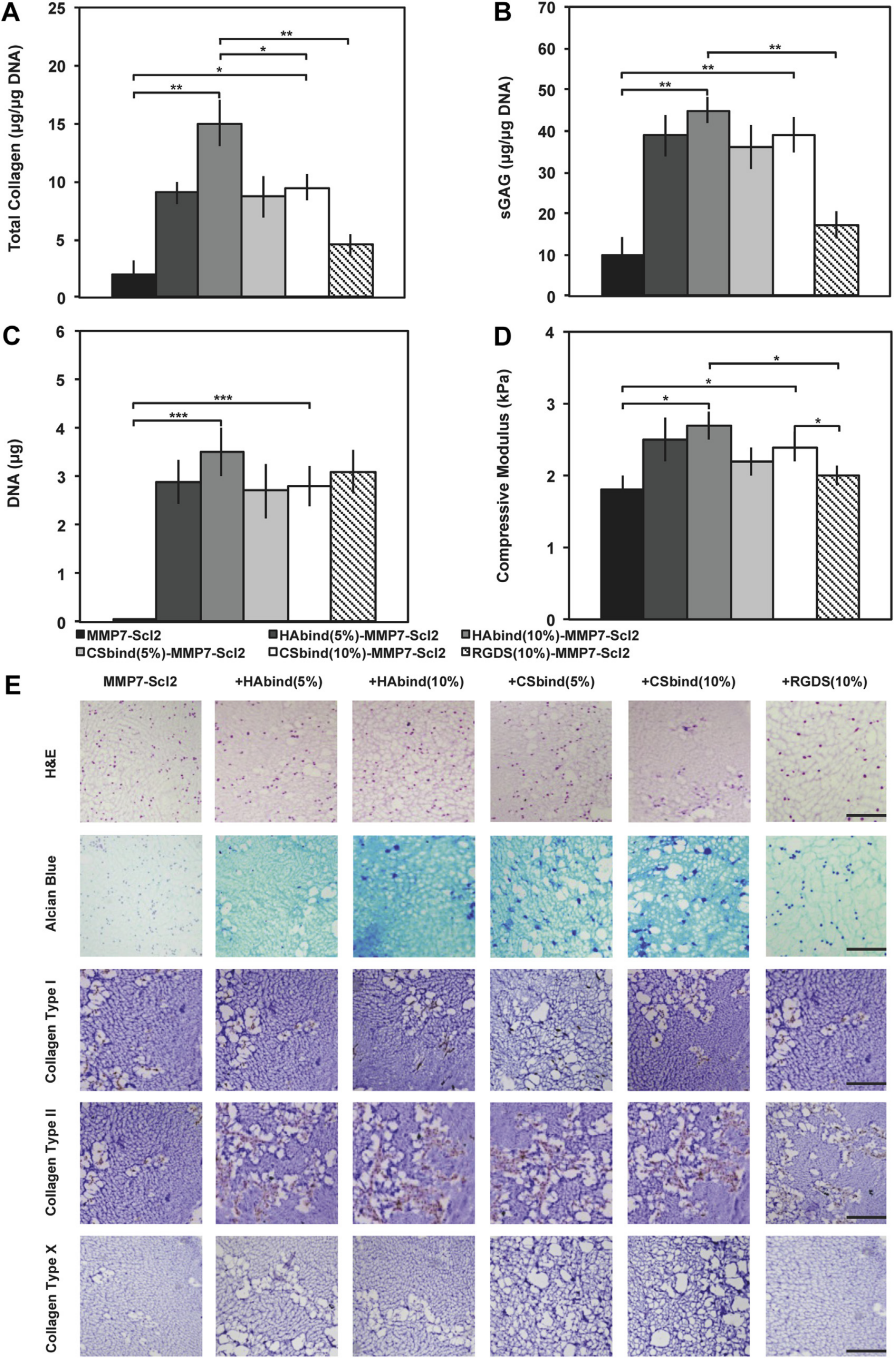
Collagen and glycosaminoglycan deposition. (A) Total collagen content in hydrogels cultured over 4 weeks quantified using a hydroxyproline assay. (B) sGAG content in hydrogels cultured over 4 weeks quantified using a Blyscan Kit. (C) DNA content per construct in hydrogels cultured over 4 weeks quantified using a PicoGreen^®^ Kit. (D) Confined elastic modulus of compression of hydrogels compressed to 10% strain at 0.5% strain/min after 4 weeks of *in vitro* culture. Values are represented as means ± SD. *P < 0.05, **P < 0.01, ***P < 0.001 (n = 3 for each donor, three different bone marrow-derived hMSC donors). (E) Histological examination of hydrogels after 4 weeks of *in vitro* culture. Hydrogels are stained with haematoxylin and eosin (H&E), alcian blue for sGAG, and by IHC for collagen type I, collagen type II and collagen type X, respectively, from top to bottom. All scale bars represent 200 μm. (For interpretation of the references to color in this figure legend, the reader is referred to the web version of this article.)

The hMSC-seeded hydrogels cultured for 4 weeks *in vitro* were also analyzed for collagen, sGAG accumulation, and DNA content. Total collagen normalized to DNA, sGAG normalized to DNA, and DNA content were highest for the HAbind(10%)-MMP7-Scl2 hydrogels compared to the other hydrogel formulations (Fig. 7A, B and C) (*P* < 0.05), further validating a positive effect of the HAbind peptide for chondrogenesis. This finding also translated to significantly higher compressive moduli for the HAbind(10%)-MMP7-Scl2 hydrogels compared to the MMP7-Scl2 and RGDS-MMP7-Scl2 controls (Fig. 7D) (*P* < 0.05). This difference in compressive moduli may be linked to the HAbind(10%) hydrogel having the highest total collagen content and collagen-to-sGAG ratio compared to the MMP7-Scl2 and RGDS-MMP7-Scl2 samples (Fig. S10) (*P* < 0.05). To a slightly lesser extent, CSbind-MMP7-Scl2 hydrogels also resulted in significantly increased total collagen and sGAG contents, as well as increased compressive moduli compared to the MMP7-Scl2 and RGDS-MMP7-Scl2 hydrogels (*P* < 0.05). The presence of the HAbind and CSbind peptides within the hydrogels may have also led to endogenous recruitment and retention of HA and CS, respectively [10], [19]. These hydrophilic, highly charged GAGs are known to aid in tissue hydration and contribute to the compressive strength in native articular cartilage [51].

Histological evaluation of the hydrogels showed a uniform distribution of cells and ECM production for all hydrogel formulations after 4 weeks of *in vitro* culture (Fig. 7E). Extensive sGAG accumulation (Alcian Blue staining) and distribution around encapsulated cells was more pronounced in the HAbind-MMP7-Scl2 and CSbind-MMP7-Scl2 hydrogels compared to the MMP7-Scl2 and RGDS-MMP7-Scl2 controls with less matrix production. Immunohistochemical analysis of collagen type II also correlated with the gene expression data for all hydrogels. Collagen type II was present in all hydrogel formulations and was notably higher in the HAbind-MMP7-Scl2 and CSbind-MMP7-Scl2 hydrogels compared to the MMP7-Scl2 and RGDS-MMP7-Scl2 controls. This corresponded with the total collagen and sGAG content shown in Fig. 7A and B, respectively. Staining for collagen type I was low in all hydrogel formulations while staining for collagen type X, an indicator of chondrocyte hypertrophy [9], was negative in all hydrogel formulations. This supports our previous findings and indicated that the hMSCs were differentiating towards chondrogenesis in the GAG-binding hydrogels.

### 3.6 Cell-mediated hydrogel degradation

**Fig. 8:**
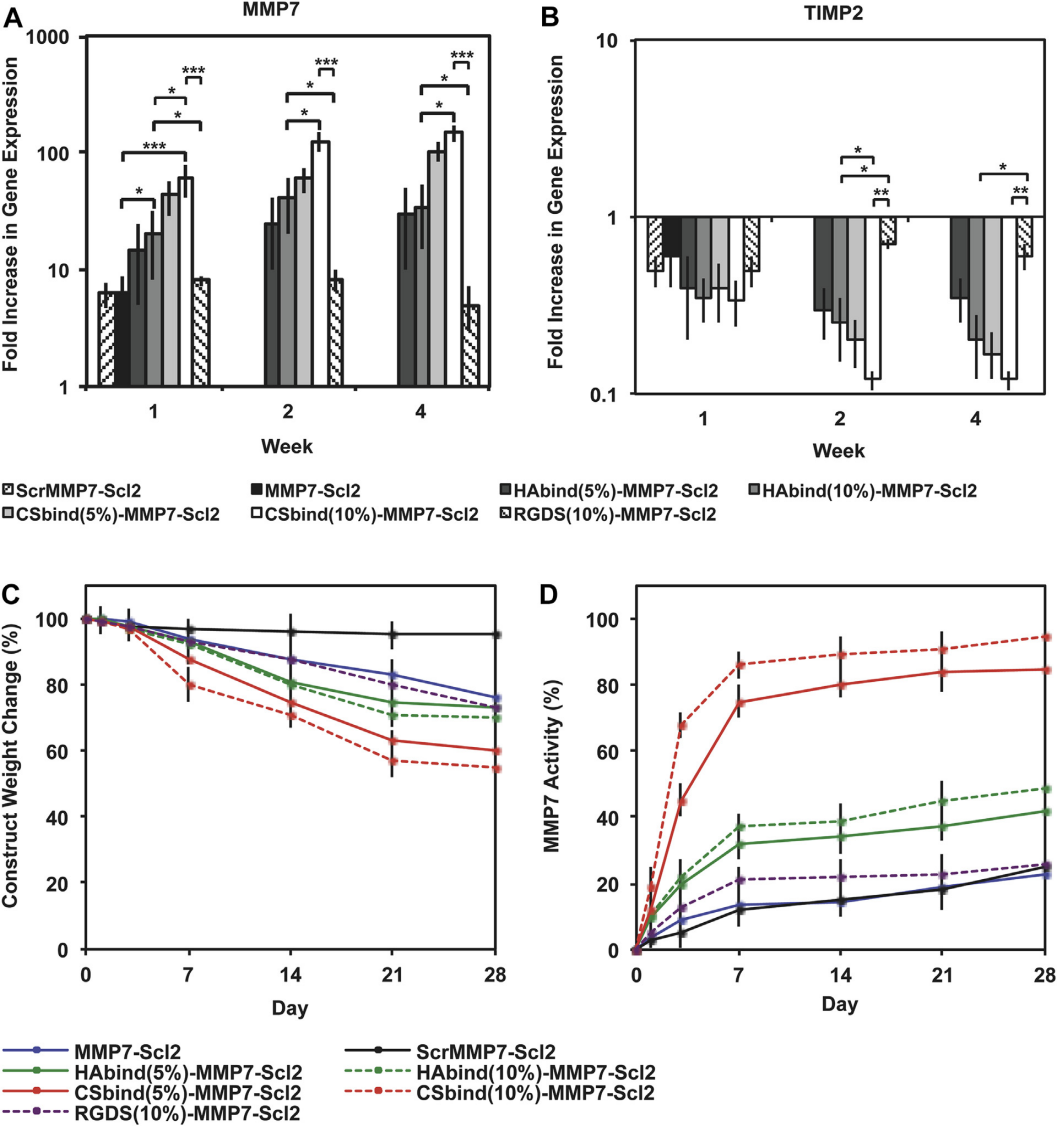
Confirmation of MMP7 as an enzyme for bioresponsive hydrogels. (A) MMP7 and (B) TIMP2 gene expression for hMSCs encapsulated in hydrogels over 4 weeks *in vitro*, as measured by qPCR and analyzed using the ΔΔCT method, represented as a fold difference relative to undifferentiated hMSCs (calibrator) prior to encapsulation and normalized to GAPDH. Values are represented as means ± SD. *P < 0.05, **P < 0.01, ***P < 0.001 (n = 3 for each donor, three different bone marrow-derived hMSC donors). Degradation of hMSC-seeded hydrogels correlated to MMP7 activity. (C) Degradation characterized by dry weight change of the construct over time. Degradation was normalized to dry weight at day 0. (D) MMP7 activity quantified in hydrogels cultured over 4 weeks *in vitro* using an MMP7 fluorogenic substrate assay. MMP7 activity was normalized to fluorescence output at day 0. Values are represented as means ± SD (n = 3 for each donor, three different bone marrow-derived hMSC donors).

As the HAbind and CSbind peptides had a significant effect on the differentiation behavior of the hMSCs, we expected to observe an effect on the gene expression and activity of MMP7 since matrix remodeling is linked to chondrogenesis. The gene expression of MMP7 (Fig. 8A) was significantly increased in cells encapsulated within the CSbind-MMP7-Scl2 and HAbind-Scl2-MMP7 hydrogels compared to other materials (CSbind-MMP7-Scl2; *P* < 0.001, HAbind-MMP7-Scl2; *P* < 0.05). Furthermore, it was significantly upregulated at all time points for the CSbind(10%)-MMP7-Scl2 hydrogels compared to the HAbind-MMP7-Scl2 (*P* < 0.05), RGDS-MMP7-Scl2 (*P* < 0.001), and MMP7-Scl2 (*P* < 0.001) hydrogels. The gene expression of TIMP2, a known inhibitor of MMP7 [52], exhibited a reverse trend with the lowest levels measured in CSbind(10%)-MMP7-Scl2 hydrogels (Fig. 8B). CS is a highly sulfated GAG that is thought to enhance the autolytic molecular activation of proMMP7 and activity of active MMP7 [47]. The CS-binding peptide may be recruiting and/or interacting with endogenous CS produced by the differentiating hMSCs to promote the gene expression and activity of MMP7 [10], [47]. The weight loss observed for the CSbind-MMP7-Scl2 hydrogels also supports this difference in MMP7 gene expression and activity (Fig. 8C). In contrast to the acellular degradation kinetics that showed similar degradation rates to the MMP7-Scl2 control in the presence of exogenous MMP7 for both the HAbind-MMP7-Scl2 and CSbind-MMP7-Scl2 hydrogels, the degradation of the cell-seeded CSbind-MMP7-Scl2 hydrogels were significantly greater (45 ± 4.6%), with higher activity of endogenous MMP7 after 4 weeks *in vitro* compared to the HAbind-MMP7-Scl2, MMP7-Scl2, and RGDS-MMP7-Scl2 hydrogels (Fig. 8D) (*P* < 0.05). Initially, the construct weight reduction was significantly higher for the CSbind-MMP7-Scl2 hydrogels compared to the other hydrogels (*P* < 0.05), suggesting that it was primarily mediated by the degradation of the hydrogel via endogenous MMP7 rather than the accumulation of matrix. This positively correlated with the MMP7 gene expression and activity data where MMP7 gene expression and activity were significantly higher for the CSbind-MMP7-Scl2 hydrogels compared to the other hydrogels. Taken together, the chondrogenesis and biodegradation results suggest the potential to combine the HAbind and CSbind peptides within a single hydrogel system using the Scl2 protein backbone to impart improved control over the rates of matrix accumulation and hydrogel degradation.

The compressive moduli of the cell-seeded HAbind-MMP7-Scl2 and CSbind-MMP7-Scl2 hydrogels (Fig. 7D) did not change significantly compared to their acellular counterparts (Fig. 3C) after 4 weeks despite the significant construct weight reduction. Our data showed that both hydrogels accumulated a significantly greater amount of sGAG matrix compared to the MMP7-Scl2 and RGDS-MMP7-Scl2 hydrogels after 4 week *in vitro* (Fig. 7B), which likely contributed to the compressive moduli of the hydrogels. Interestingly, the weight of the HAbind-MMP7-Scl2 and CSbind-MMP7-Scl2 constructs was significantly lower compared to the MMP7-Scl2 and RGDS-MMP7-Scl2 constructs. However, taken together with the sGAG data, these results suggest the higher sGAG accumulation in the HAbind-MMP7-Scl2 and CSbind-MMP7-Scl2 constructs may be contributing to the compressive moduli. By Week 4, there was a plateau in the construct weight change (Fig. 8) for the HAbind-MMP7-Scl2 and CSbind-MMP7-Scl2 hydrogels compared to the MMP7-Scl2 and RGDS-MMP7-Scl2 hydrogels, suggesting a balance between the degradation of the hydrogel and the accumulation of matrix that is supported by the total collagen and sGAG accumulation data (Fig. 7). In addition, the MMP7 concentration used in the acellular degradation studies was considerably higher (30 ng/mL) than physiological levels (0.3 ng/mL) [44] to accelerate the overall degradation. At physiological levels, it is expected that the construct weight change for all hydrogel formulations would be more strongly affected by the matrix accumulation than the hydrogel degradation over a longer incubation period. Hydrogels cross-linked via MMP-cleavable peptides have demonstrated cell-mediated degradation that plays a role in cellular processes such as chondrogenesis and differentiation [13], [20], [53], [54]. In our work, incorporating GAG-binding motifs within the hydrogels is shown to regulate MMP7 gene expression and activity and, arguably, the chondrogenic differentiation of encapsulated hMSCs.

The combination of MMP-cleavable cross-links and the bioactive components introduces the possibility to mediate inherent biological processes to guide the dynamics of tissue regeneration and remodeling. Incorporating varying ratios of the HAbind and CSbind peptide motifs in a single hydrogel can be used to tune the kinetics of chondrogenesis and ECM accumulation to match the rate of cell-mediated hydrogel degradation. In addition, the modularity allows for other MMP-sensitive peptides and bioactive sequences to be added easily through tethering or site-directed mutagenesis to the collagen-like protein backbone to recreate the complexity of the ECM and mimic zone-specific microenvironments. This is particularly useful when optimizing the hydrogel for *in vivo* translation where the potentially inflammatory and native ECM interactions could induce unintended protease activities. This novel collagen-like protein-based design provides a platform that can also be readily adapted with multiple and selected peptide sequences for desired applications in other areas of regenerative medicine.

## 4 Conclusions

We have designed and developed novel biodegradable hydrogels based on recombinant collagen-mimetic Scl2 proteins that can be easily tailored to recreate the biochemical microenvironment of articular cartilage. The blank slate Scl2 protein was tethered with GAG-binding peptides specific for HA and CS and cross-linked into an injectable hydrogel with MMP7-sensitive peptide for cell-mediated degradation. The presence of the GAG-binding peptides significantly enhanced the chondrogenic characteristics of the constructs encapsulated with hMSCs. Specifically, the HAbind-MMP7-Scl2 hydrogels directed the highest increase in the gene expression of COL2A1, ACAN, and SOX9 by hMSCs leading to the greatest total collagen and sGAG accumulation. The CSbind-MMP7-Scl2 hydrogels had the greatest impact on the gene expression and activity of endogenous MMP7 that correlated with the greatest change in the articular cartilage deposition/degradation ratio. Taken together, our hydrogels demonstrate a high degree of modularity, highlighting the potential to incorporate multiple peptides to recreate the complex dynamics of native ECM, and introduce a minimally invasive approach to promote chondrogenesis with host MSCs.
