# Supplementary material for: Collagen-mimetic peptide-modifiable hydrogels for articular cartilage regeneration
Source: Biomaterials. 2015 Jun;54:213–25. doi: 10.1016/j.biomaterials.2015.02.079 (PMC4416732; doi:10.1016/j.biomaterials.2015.02.079)
Supplement: Supplementary file 1 [file mmc1.pdf]

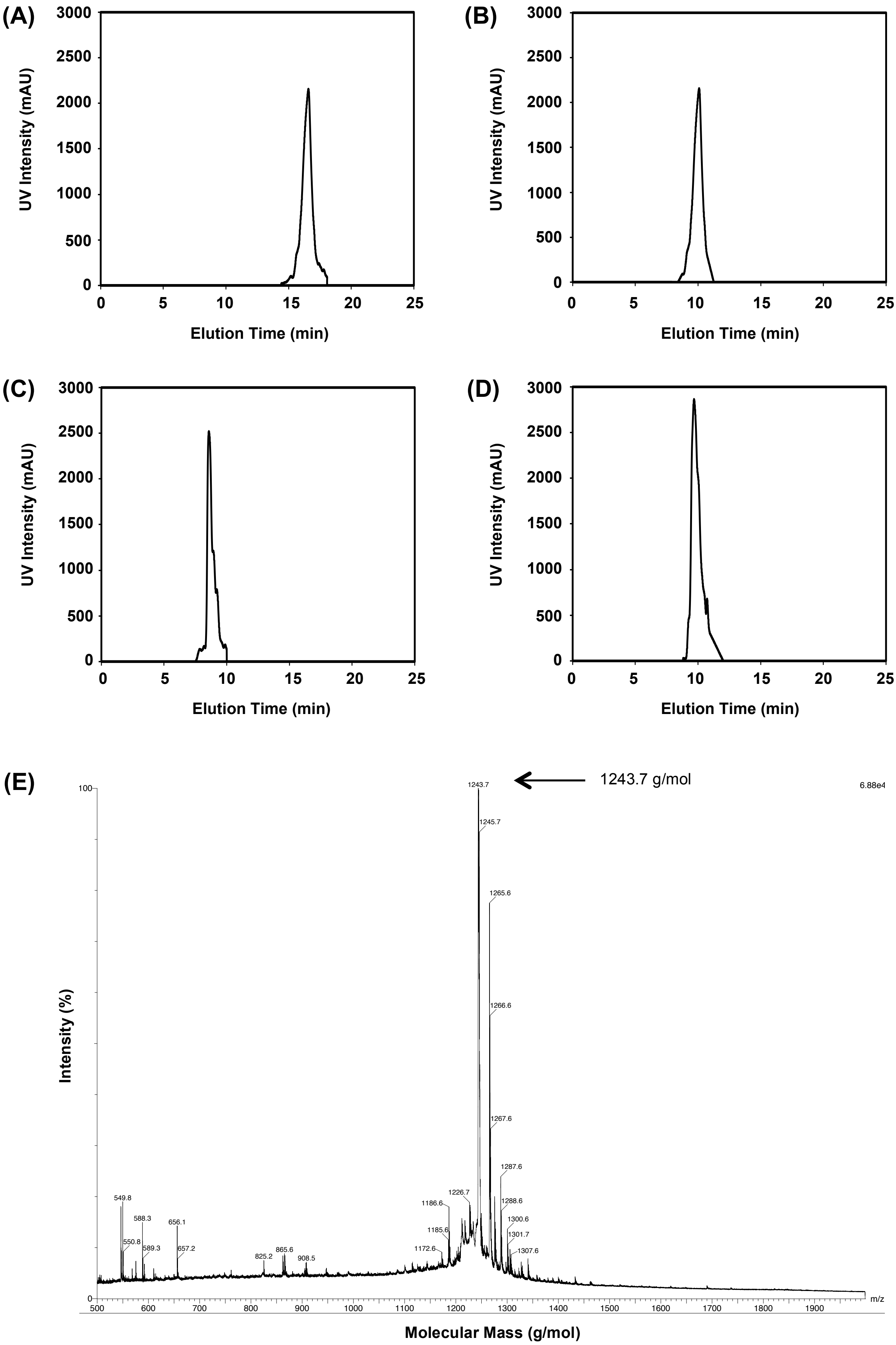
(A)
(B)
(C)
(D)
UV Intensity (mAU)
Elution Time (min)
0
500
1000
1500
2000
2500
3000
0
5
10
15
20
25
(E)
1243.7 g/mol
6.88e4
Intensity (%)
Molecular Mass (g/mol)
m/z
100
0
500
600
700
800
900
1000
1100
1200
1300
1400
1500
1600
1700
1800
1900
1243.7
1245.7
1265.6
1266.6
1267.6
1287.6
1288.6
1300.6
1301.7
1307.6
1226.7
1186.6
1185.6
1172.6
549.8
550.8
588.3
589.3
656.1
657.2
825.2
865.6
908.5

(F)

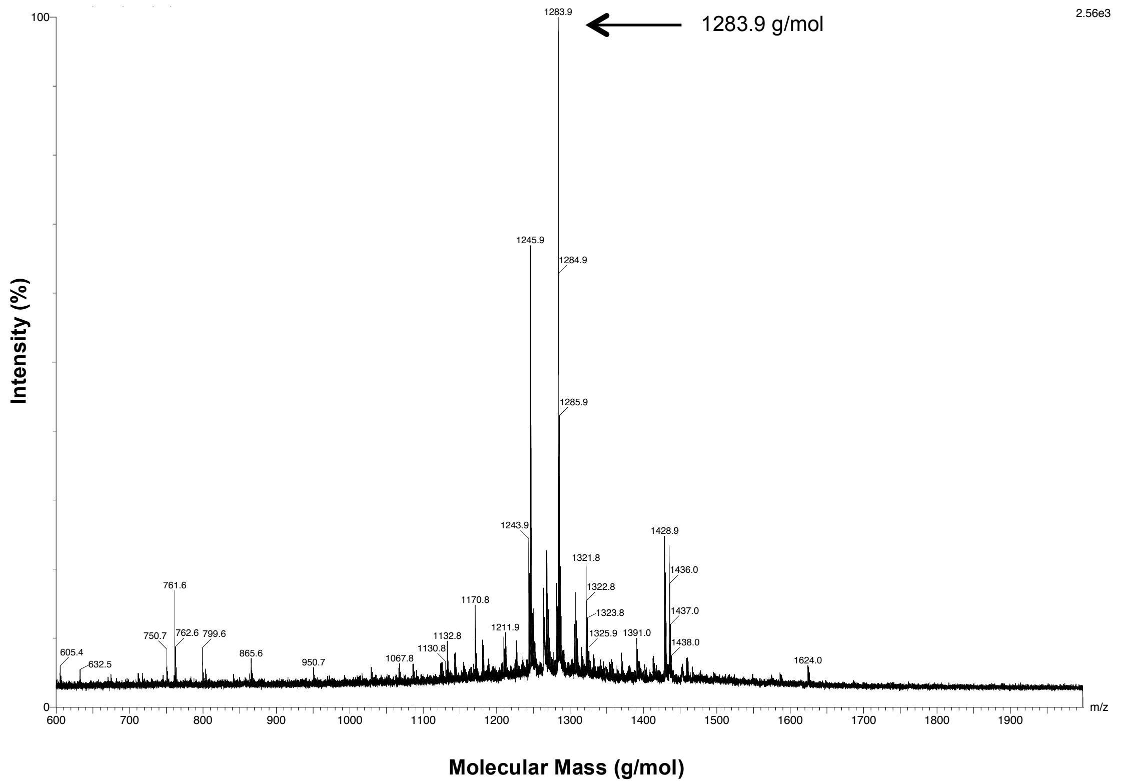
1283.9 g/mol
2.56e3
Intensity (%)
Molecular Mass (g/mol)
m/z
1283.9
1245.9
1284.9
1285.9
1243.9
1428.9
1321.8
1436.0
1322.8
1323.8
1437.0
1170.8
1211.9
1325.9
1391.0
1438.0
1132.8
1130.8
1067.8
950.7
865.6
761.6
750.7
762.6
799.6
605.4
632.5
1624.0

(G)

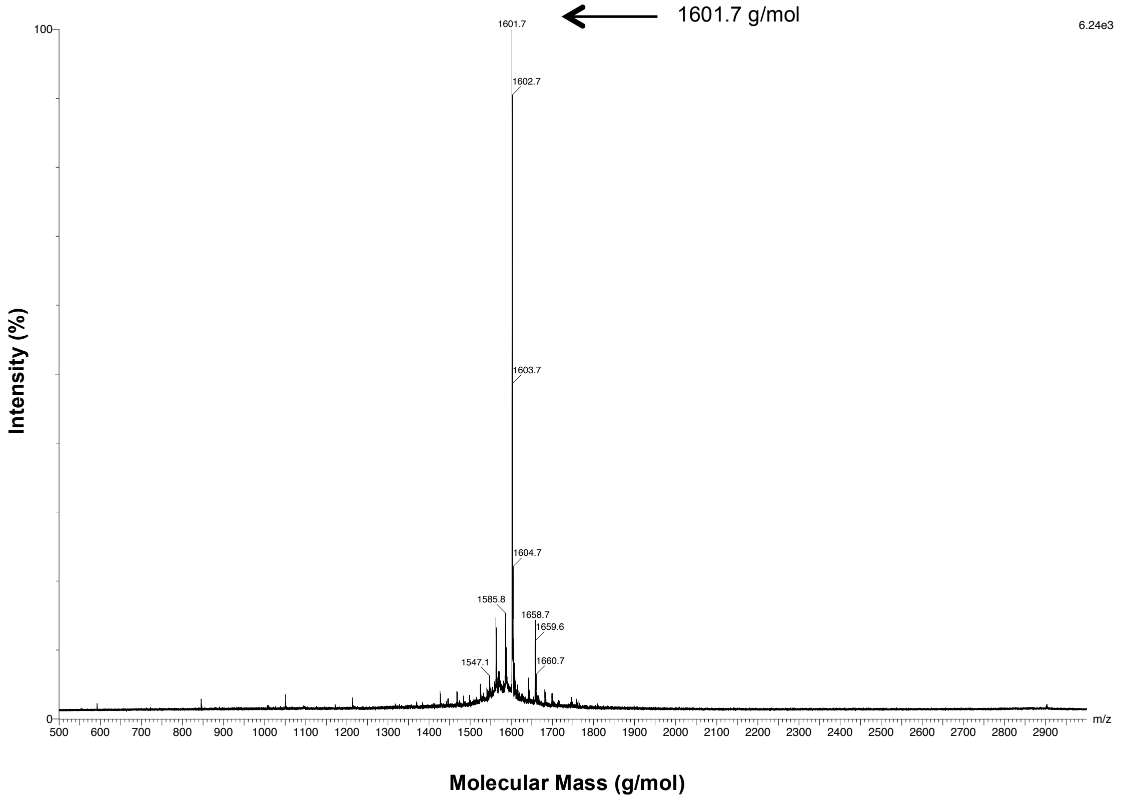
1601.7 g/mol
6.24e3
Intensity (%)
Molecular Mass (g/mol)
m/z
1601.7
1602.7
1603.7
1604.7
1585.8
1658.7
1659.6
1547.1
1660.7

(H)

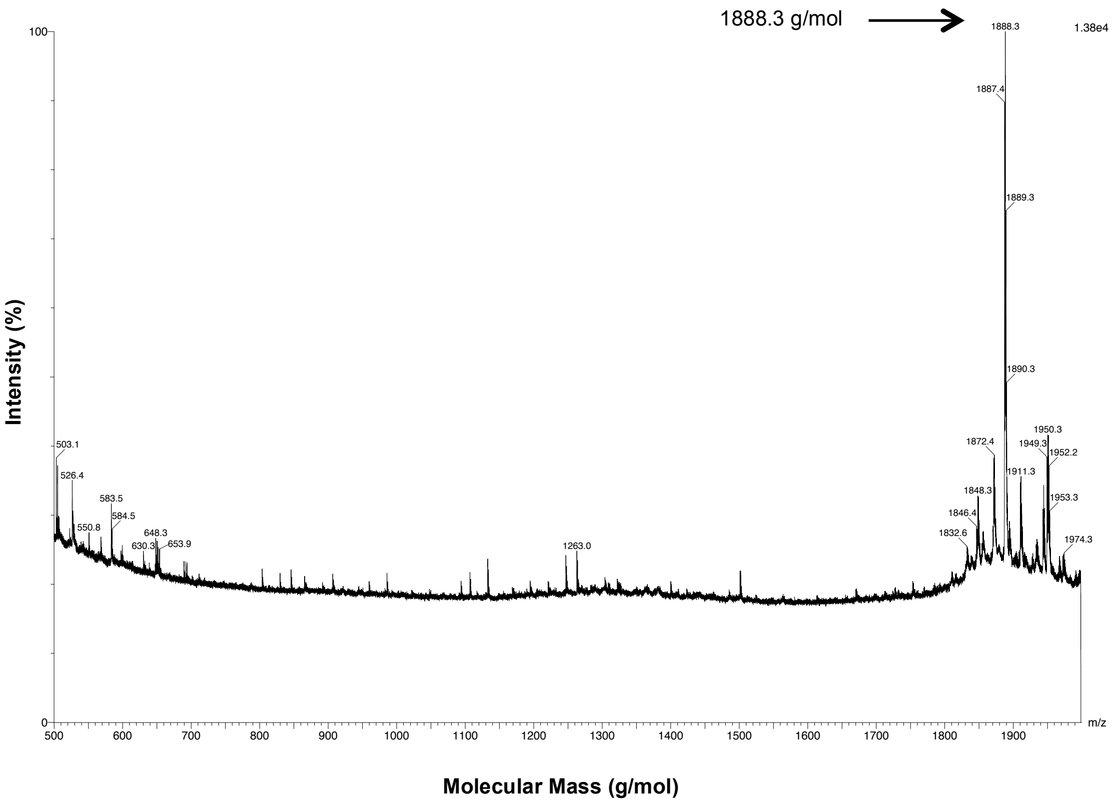

Fig. S1. Representative reversed phase HPLC chromatograms and MALDI spectra of (A, E) MMP7 (MW:1243.7 g/mol), (B, F) ScrMMP7 (MW:1283.9 g/mol), (C, G) HAbind (MW:1601.7 g/mol) and (D, H) CSbind (MW:1888.3 g/mol) peptides, respectively.

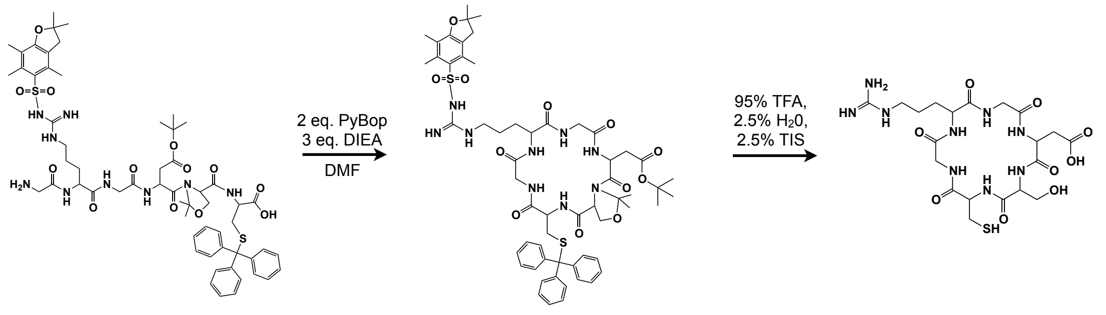

(1) (2) (3)

Fig. S2. Synthesis scheme for cyclic GRGDSC showing the linear protected peptide GR(Pbf)GD(Otbu)S(psiMe,Mepro)C(Trt) (1) converted to a cyclized protected peptide (2) then cleaved to form cyclic GRGDSC (3).

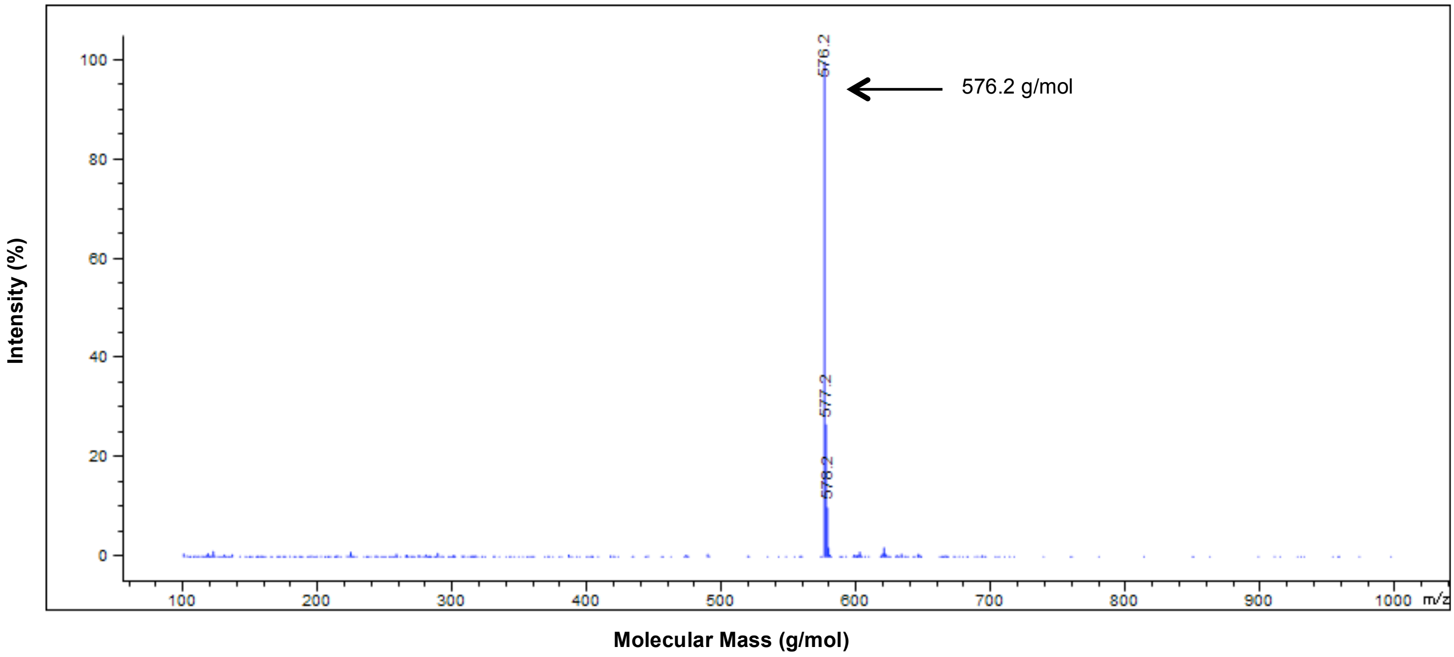

Fig. S3. ESI of the purified cyclic GRGDSC (MW 576.2 g/mol).

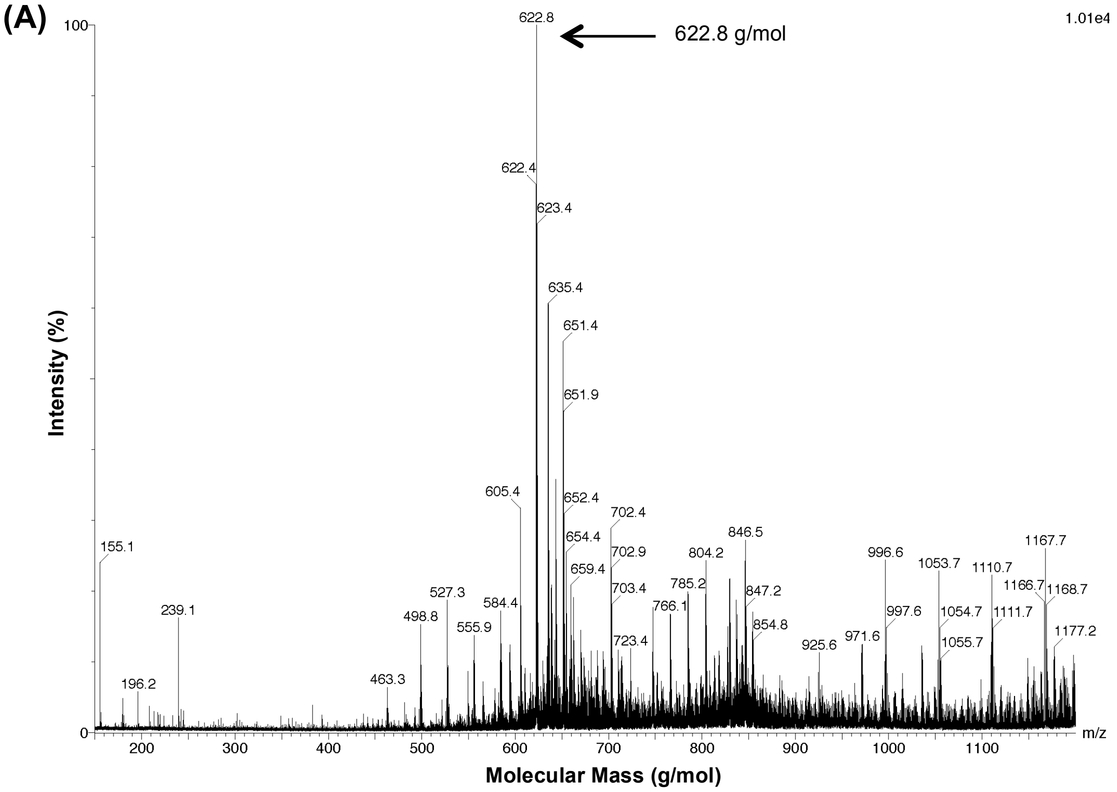

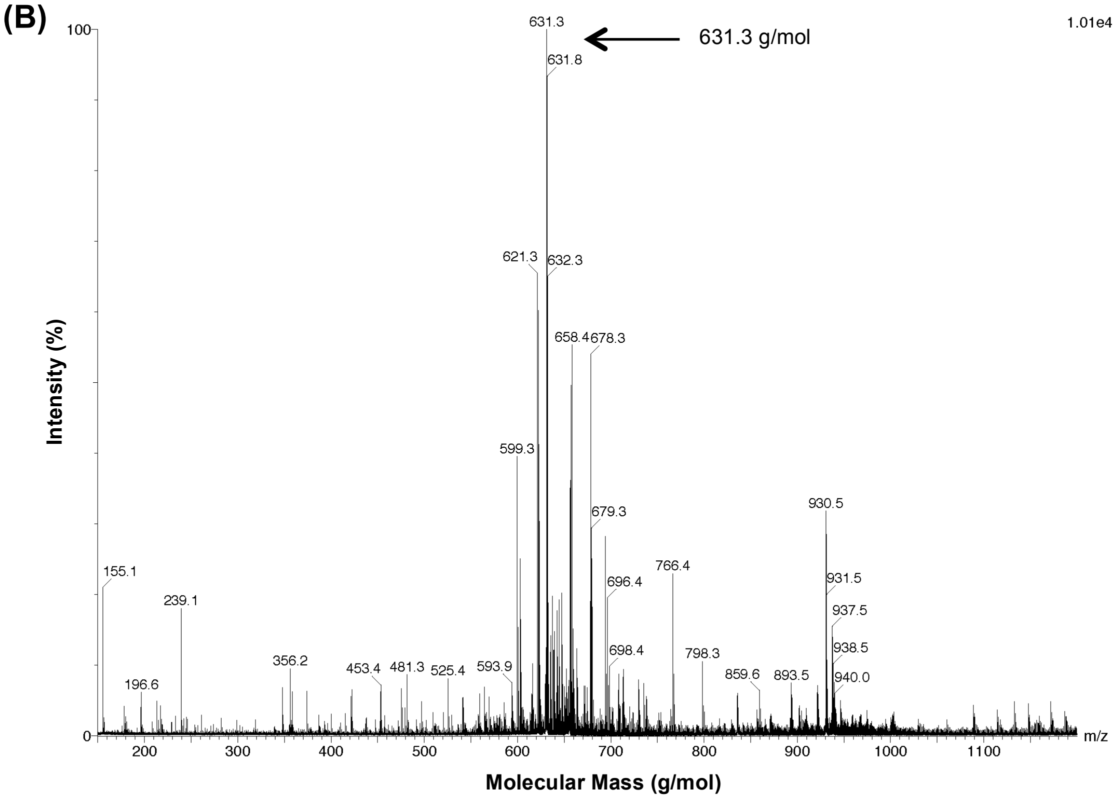

Fig. S4. Representative LCMS-ESI spectra of MMP7 peptide obtained at (A) M/2 fragment (622.8 g/mol) from chromatogram without and (B) M fragment (631.8 g/mol) from chromatogram with exogenous recombinant human MMP7, confirming cleavage at the expected site on the peptide between glutamic acid and leucine.

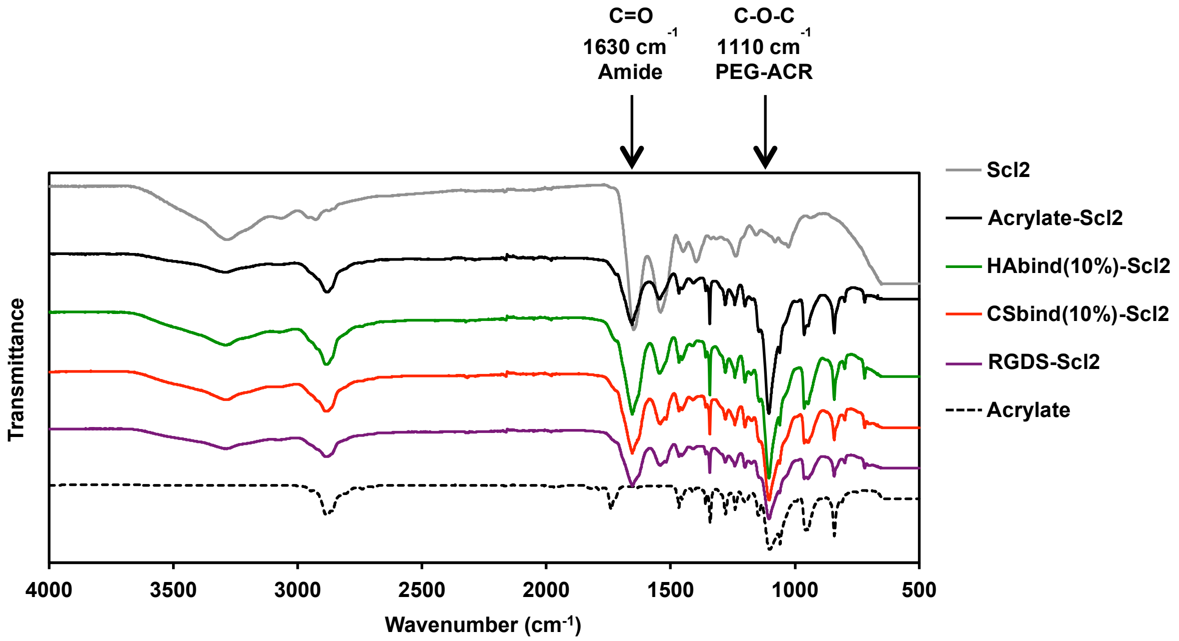

Fig. S5. Representative FTIR spectra of functionalized Scl2 proteins confirming the conjugation of Scl2 with linker, HAbind, CSbind and RGDS peptides.

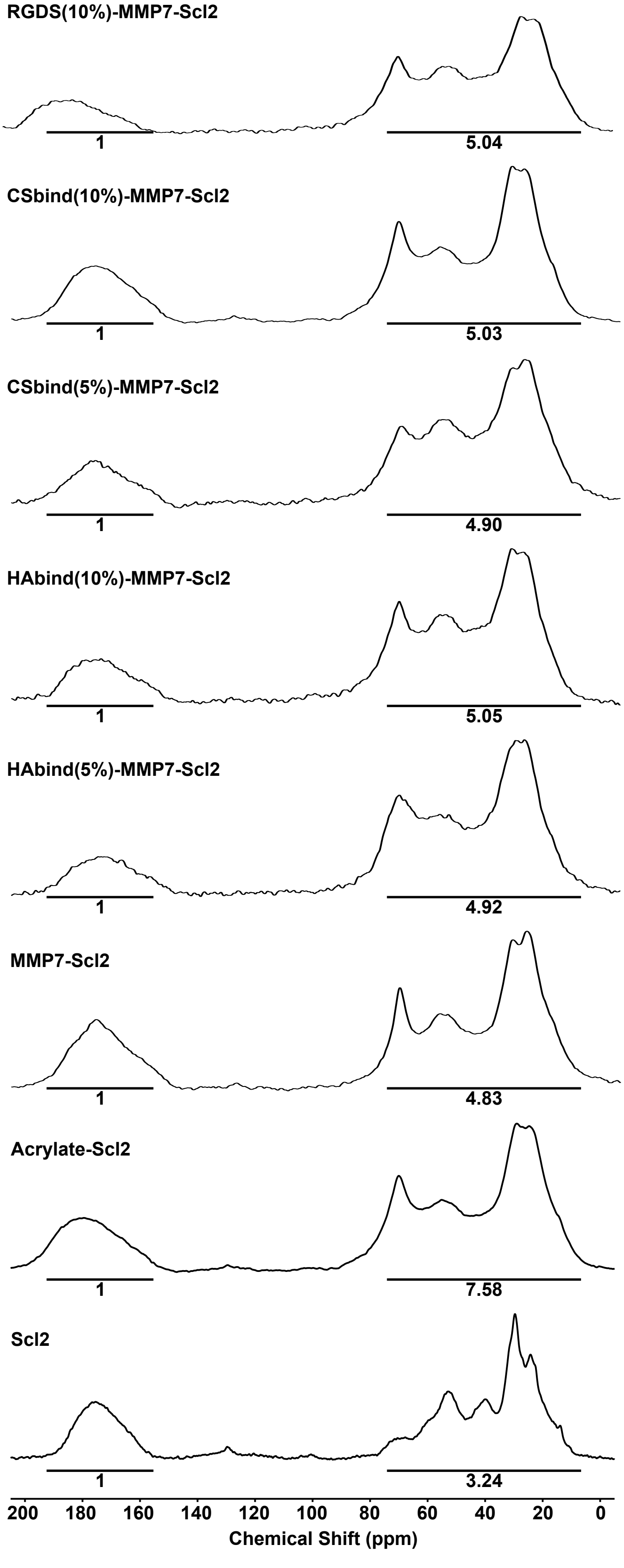

Fig. S6. Representative cross-polarization magic-angle spinning (CP MAS) solid-state ^{13}C-NMR spectra of functionalized Scl2 proteins quantifying the conjugation of Scl2 with linker, MMP7, HAbind, CSbind and RGDS peptides. The measured degree of conjugation was determined from the respective spectra.

Table. S1.

Degree of conjugation of functionalized Scl2 hydrogels. Degree of conjugation of functionalized Scl2 proteins with linker, MMP7, HAbind, CSbind and RGDS peptides was determined using cross-polarization magic-angle spinning (CP MAS) solid-state ^{13}C-NMR. The measured degree of conjugation was determined from the respective spectra by calculating the ratio of the integrated carbonyl peak and the aliphatic peak followed by their linear combination with the acrylate-Scl2 sample.

Sample	Component	Expected Degree of Conjugation (%)	Actual Degree of Conjugation (%)
Scl2	N/A	N/A	N/A
acrylate-Scl2	acrylate	33.3	31.6
MMP7-Scl2	MMP7	90	86.1
HAbind(5%)-MMP7-Scl2	HAbind	5	4.8
HAbind(10%)-MMP7-Scl2	HAbind	10	9.6
CSbind(5%)-MMP7-Scl2	CSbind	5	4.6
CSbind(10%)-MMP7-Scl2	CSbind	10	9.7
RGDS(10%)-MMP7-Scl2	RGDS	10	9.6

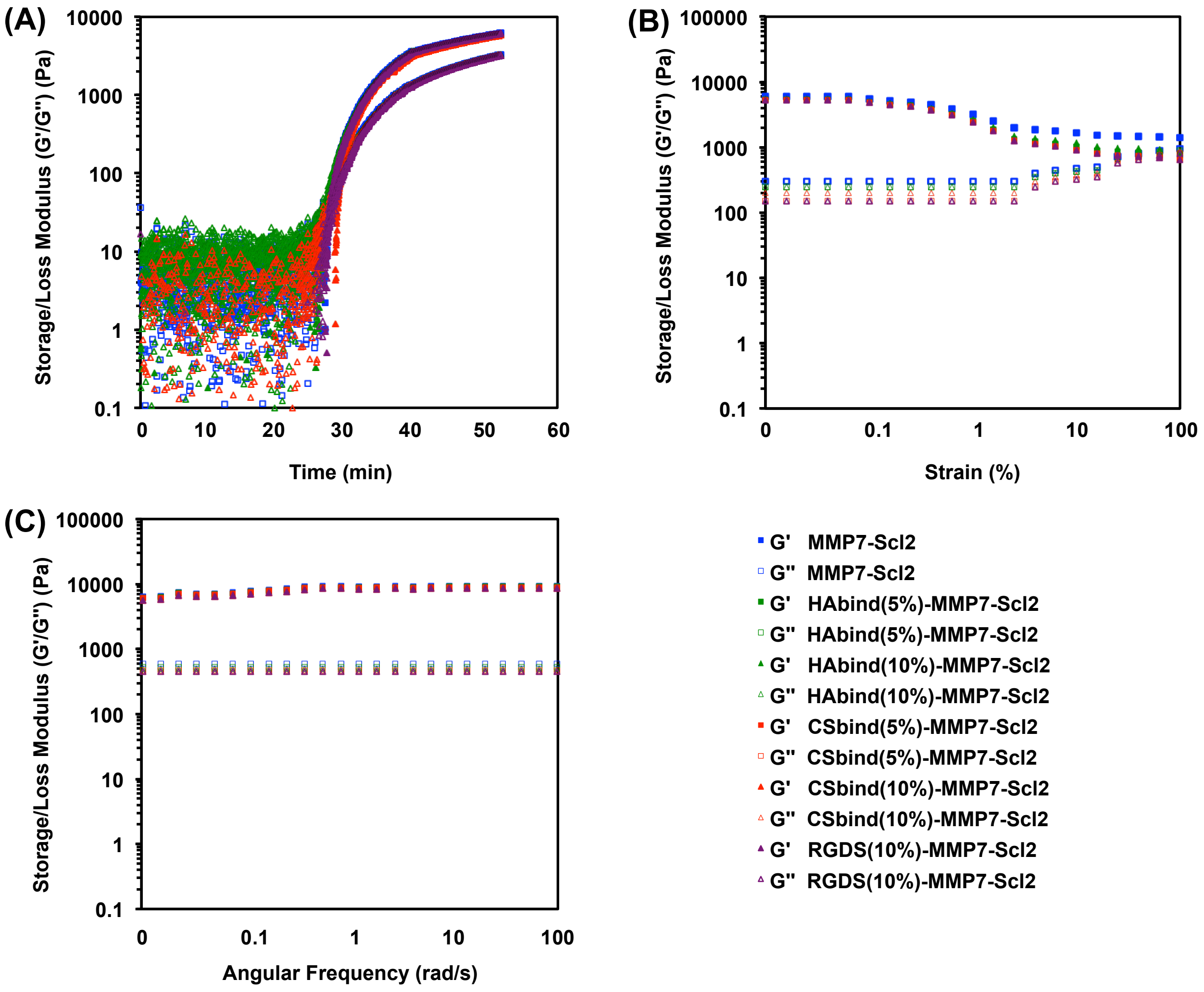

Fig. S7. Rheological properties of functionalized Scl2 hydrogels. (A) Time to gelation determined using respective time to gelation data in (C) at a temperature of 37 °C, angular frequency of 6.28 rad/s and strain of 0.5%. (B) Strain sweep at a temperature of 37 °C and an angular frequency of 6.28 rad/s shown as G' and G''. (C) Angular frequency sweep at a temperature of 37 °C and 0.5% strain shown as G' and G''. Values are represented as means ± SD.

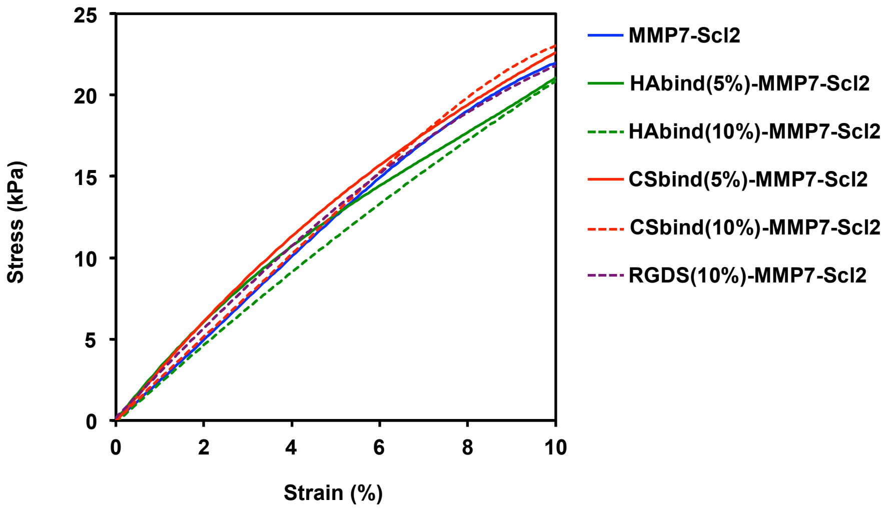

Fig. S8. Representative stress-strain curves of functionalized Scl2 hydrogels. Acellular hydrogels were compressed to 10% strain at 0.5% strain/min (n = 3).

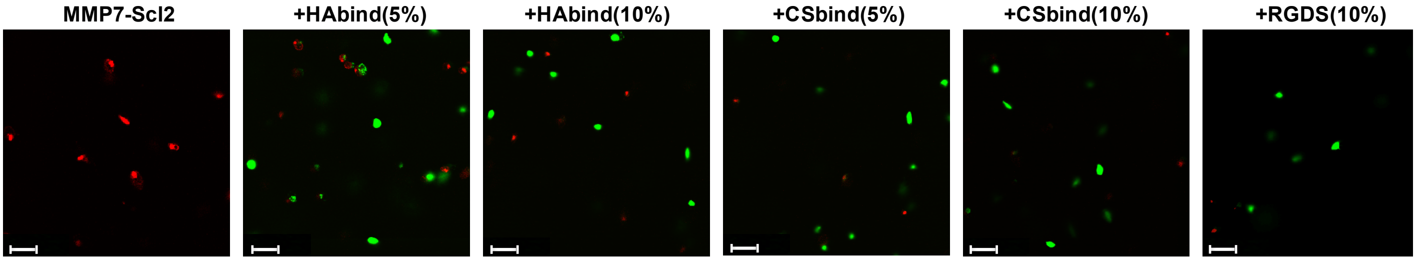

Fig. S9. hMSC viability in hydrogels cultured over 4 weeks using the LIVE/DEAD® Viability/Cytotoxicity assay. All scale bars represent 100 μm.

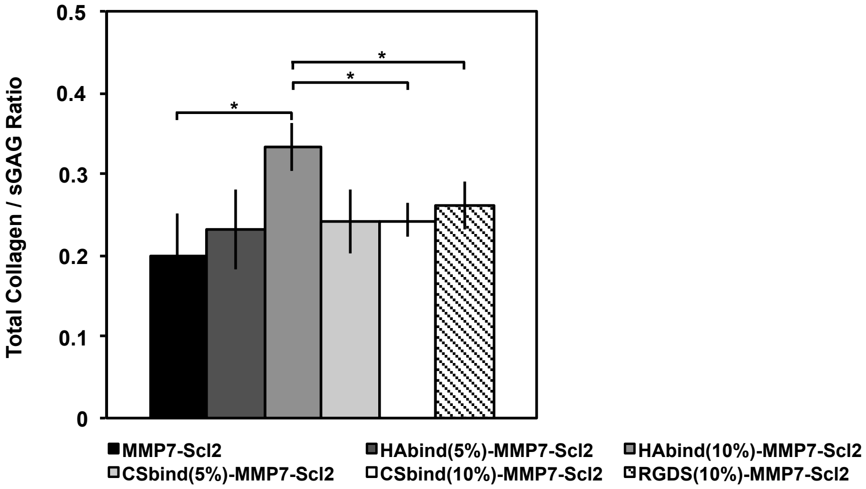

Fig. S10. Total collagen-to-sGAG ratio content quantified in hydrogels cultured over 4 weeks. Total collagen and sGAG content determined using a hydroxyproline assay and a Blyscan Kit, respectively. Values are represented as means ± SD. *$P < 0.05$ (n = 3).
